# Rotational Spectrum and Conformational Analysis of *N*-Methyl-2-Aminoethanol: Insights into the Shape of Adrenergic Neurotransmitters

**DOI:** 10.3389/fchem.2018.00025

**Published:** 2018-02-22

**Authors:** Camilla Calabrese, Assimo Maris, Luca Evangelisti, Anna Piras, Valentina Parravicini, Sonia Melandri

**Affiliations:** Dipartimento di Chimica “G. Ciamician” dell'Università, Bologna, Italy

**Keywords:** rotational spectroscopy, quantum chemical calculations, atom in molecules theory, molecular structure, hydrogen bond, *N*-methyl-2-aminoethanol, 1,2-aminoalcohols, adrenergic neurotransmitters

## Abstract

We describe an experimental and quantum chemical study for the accurate determination of the conformational space of small molecular systems governed by intramolecular non-covalent interactions. The model systems investigated belong to the biological relevant aminoalcohol's family, and include 2-amino-1-phenylethanol, 2-methylamino-1-phenylethanol, noradrenaline, adrenaline 2-aminoethanol, and *N*-methyl-2-aminoethanol. For the latter molecule, the rotational spectrum in the 6–18 and 59.6–74.4 GHz ranges was recorded in the isolated conditions of a free jet expansion. Based on the analysis of the rotational spectra, two different conformational species and 11 isotopologues were observed and their spectroscopic constants, including ^14^N-nuclear hyperfine coupling constants and methyl internal rotation barriers, were determined. From the experimental data a structural determination was performed, which was also used to benchmark accurate quantum chemical calculations on the whole conformational space. Atom in molecules and non-covalent interactions theories allowed the characterization of the position of the intramolecular non-covalent interactions and the energies involved, highlighting the subtle balance responsible of the stabilization of all the molecular systems.

## Introduction

The essential chemical and biological processes of molecular transport and recognition are determined by the shape of molecules and the weak interactive forces between them. Therefore, a study of the conformational landscapes and the non-covalent interactions in isolated systems or small molecular complexes is necessary to understand the influence of these factors on the mechanisms of transport and drug-receptor interaction (Robertson and Simons, 2001), as well as reactivity and catalysis (Sohtome and Nagasawa, 2012; Hanoian et al., 2015). High resolution spectroscopy combined with quantum chemical calculations on isolated systems has proved a valuable means to investigate the conformational behavior of molecules (Caminati and Grabow, 2009), biomolecules (Alonso and Lopez, 2015), and molecular complexes (Melandri and Becucci, 2016) in the absence of external factors such as solvent interactions and crystal packing effects. The results allow the unambiguous identification of different conformers (Vigorito et al., 2017), tautomers (Sanchez et al., 2007), isotopologues (Calabrese et al., 2015), and enantiomers (Patterson et al., 2013; Lobsiger et al., 2015) and can also be applied to the investigation of non-covalently bound molecular adducts (Calabrese et al., 2016).

In general, hydroxyl and amino groups rank among the most important hydrogen-bonding building blocks in nature. In particular, if the two functional groups are separated by a backbone of two *sp*3 carbon atoms or more, such as in the 1,2-aminoalcohols, an interesting interplay and competition between intra- and intermolecular hydrogen bonds (HB) is possible. The aminoalcohol family of compounds presents several interesting properties. From the chemical point of view, the combination of donor and acceptor groups in the same molecule stabilizes molecular conformations involving intramolecular HB either of OH···N or NH···O type (Penn and Curl, 1971) while, from a biological point of view, one can note that many natural products contain the aminoalcohol functionality (Robertson and Simons, 2001).

Among the biological examples, the 1,2-aminoalcohol chain is present both in adrenaline and noradrenaline (respectively also known as epinephrine and norepinephrine) which are important adrenergic neurotransmitters, belonging to the catecholamines' family. These compounds are of great biochemical and pharmaceutical interest because they are directly biosynthesized by human beings, regulating several functions of cardiovascular and metabolic systems, and they have a common use as drugs for vascular diseases (Patrick, 2009). The characterizations of the conformational space of adrenaline (Çarçabal et al., 2005) and noradrenaline (Snoek et al., 2003) with spectroscopic and computational methods, has been performed. Smaller analogs of adrenaline, halostachine (2-methylamino-1-phenylethanol, MAPE) and of noradrenaline (amino-1-phenylethanol, APE) have also been the object of spectroscopic and theoretical researches (Graham et al., 1999; Butz et al., 2001; Melandri et al., 2009). These studies have shown that the different conformations in both the neurotransmitters and the model systems arise from different arrangements of the aminoalcohol chain which can be folded or extended, influencing the subtle balance of non-covalent and steric interactions which govern the stability of the different geometrical arrangements.

Aminoalcohols are also interesting from an astrobiological point of view, since the simplest ones can be considered as precursors for the formation of aminoacids in the interstellar medium (Charnley, 1999). Moreover, as reported by Ehrenfreund and Charnley (2000), the evaporation on ice mantles containing ammonia, as well as methanol and ethanol, could be the origin of the nitrogen-bearing organics that are seen in star-forming regions and maybe other larger analogs that have not yet been discovered. Since molecules in space are detected through their emission spectra, and in particular because the rotational spectra can be considered as their fingerprint, laboratory spectral characterization in the microwave region can be directly used for their detection through the latest generation radiotelescopes such as ALMA (Atacama Large Millimeter/submillimeter Array).

For all of the above reasons, the 1,2-aminoalcohol family has been extensively investigated experimentally by microwave (Penn and Curl, 1971; Penn and Buxton, 1975; Widicus et al., 2003; Melandri et al., 2009), and infrared (Räsänen et al., 1982; Silva et al., 1999; Snoek et al., 2003; Liu et al., 2004; Seurre et al., 2004; Çarçabal et al., 2005; Asselin et al., 2016) spectroscopy, as well as theoretically using different levels of calculation (Räsänen et al., 1982; Silva et al., 1999; Vorobyov et al., 2002; Snoek et al., 2003; Çarçabal et al., 2005). The simplest prototype of this interesting class of molecules is 2-aminoethanol (AE). Experimental (Penn and Curl, 1971; Räsänen et al., 1982; Widicus et al., 2003; Asselin et al., 2016) theoretical (Vorobyov et al., 2002) and combined (Silva et al., 1999) studies have shown that the preferred conformation of the isolated molecule is folded with the amino group acting as the hydrogen acceptor, and that when AE is involved in 1:1 molecular complexes either with water (Tubergen et al., 2003), ammonia (Melandri et al., 2010b), or forms a dimer (Asselin et al., 2016), the other moiety is inserted into the intramolecular HB, causing a distortion of the geometry with respect to isolated AE.

In this work, first we investigate the effect of replacing one amino hydrogen by a methyl group, studying the rotational spectrum of *N*-methyl-2-aminoethanol (CH_3_-NH-CH_2_-CH_2_-OH, from now on indicated as MAE) in supersonic expansion. Despite the fact that the rotational spectrum of MAE was studied before (Penn and Buxton, 1975) the low accuracy of the measurements led us to take up again its analysis. In particular, the decision to deepen the study of MAE and its conformational space is twofold: in the first place, MAE is present as a side chain in important biological molecules such as adrenaline, thus a more detailed study can point out results that can better describe the interactions present in the isolated chain, secondly MAE has been postulated as a precursor of aminoacid formation and a better spectral characterization could help its detection in space.

In the room temperature spectrum of MAE, two conformers were observed, their dipole moment measured and their relative energy difference [1.9 (3) kJ mol^−1^], estimated from intensity measurements (Penn and Buxton, 1975). Regarding our new experimental investigation, two paths were followed: in order to better characterize the spectroscopic signatures of the molecule and extend the investigation to astrophysically useful regions the millimeter-wave range (59.6–74.4 GHz/5.03–4.03 mm) was explored, while the hyperfine structure of MAE's rotational spectrum due to the nuclear quadrupole coupling and the methyl group internal rotation was analyzed exploiting the extremely high resolution of the Molecular Beam Fourier Transform Microwave (MB-FTMW) technique based on the design by Balle and Flygare (1981). The detailed structural information obtained is used to benchmark the results of quantum chemical calculations, which are extended to model molecular systems such as AE, APE, and MAPE, adrenaline and noradrenaline to highlight the changes which occur when the side chain is different (AE) or it is linked to a phenyl (APE, MAPE) or a cathecol ring (adrenaline or noradrenaline). Atom in molecules and non-covalent interactions are well suited theories used for the examination of the nature of the different HBs bonds implicated in the stabilization of these model systems, allowing a comparison of the energies involved in the subtle forces balance in each molecule.

## Materials and methods

### Experimental details

The millimeter-wave spectrum (59.6–74.4 GHz/5.03–4.03 mm) was recorded with the Free-Jet Absorption Milli-Meter Wave spectrometer (FJ-AMMW); the basic design of which has been described previously (Calabrese et al, 2013) MAE, purchased from Sigma Aldrich (99%) and used without further purification, was vaporized at a temperature of 313 K in a stream of helium at a pressure of 45 kPa and expanded to about 0.05 kPa through a 0.35 mm diameter nozzle, held 5 K above the vaporization temperature. The bi-deuterated and both mono-deuterated species (OD-ND, OH-ND, and OD-NH, respectively) were formed by flowing D_2_O over the sample. Under these conditions, the post-expansion rotational temperature was about 10 K and no evidence of thermal decomposition was observed. Electric fields up to 750 V cm^−1^ were used to maximize the degree of Stark modulation. Lines separated by more than 300 kHz are resolvable and the estimated accuracy of the frequency measurements is better than 50 kHz.

Spectra in the range 6.5–18 GHz were obtained using a Molecular Beam Fourier Transform Microwave (MB-FTMW) spectrometer described previously (Caminati et al., 2016). The same MAE sample used for the FJ-AMMW experiment, was used without further purification. For this experiment, the sample was heated to about 75°C ahead of the nozzle and a stream of helium, used as carrier gas, at a pressure ≈200 kPa was flowed over it. The mixture was then expanded through a pulsed supersonic nozzle with a diameter of 0.5 mm. The nozzle was placed in the center of one mirror of the confocal resonation cavity and the expansion was coaxial with the chamber and with pulses of microwave radiation. The spectrum was determined after a Fourier transformation of 8 k data-point time domain signal. Every transition appears as a Doppler doublet and the mean of the two frequency is taken as the true transition frequency. The accuracy of the measurements is estimated to be better than 5 kHz and the resolution better than 7 kHz.

### Computational details

Molecular geometry optimizations are used to predict molecular equilibrium properties and conformational preferences from the potential energy surface (PES). The resulting information on rotational constants, dipole moment components, relative energies, methyl internal rotation barriers and quadrupole coupling constants are used to predict the rotational spectra and are essential for the conformational assignment. In order to obtain reliable conformational energies and molecular properties, both *ab initio* and density functional theory (DFT) methods are used to assist the assignment of the rotational spectra.

In the case of *ab initio* methods, the Møller-Plesset (Møller and Plesset, 1934; Cremer, 2011) model gives a good balance between accuracy and computational cost for spectroscopic purposes. These calculations are post-Hartree-Fock methods which explicitly introduce electron correlation through perturbation theory, usually, as in this case, up to second order (MP2). Depending on the size of the molecules, the computational costs increase quickly with the MP2 method and for this reason the DFT calculations are performed. Indeed, these kinds of methods are widely used due to the good efficiency-computational cost ratio compared to the post-Hartree-Fock ones. The B3LYP functional (Lee et al., 1988) is the one used in our spectroscopic studies, because it reproduces satisfactorily the chemical parameters required. All theoretical calculations are run with the Gaussian 09 (Frisch et al., 2013) program package. For all the optimized structures, vibrational frequency calculations were performed in the harmonic approximation to check whether all of those are real minima.

To quantify the energy related to HBs, the topology of the theoretical electron densities was analyzed with the Multiwfn program (Lu and Chen, 2012) which is based on the *Atoms in Molecules Theory* (AIM) (Bader, 1990). Complementary information was also achieved visualizing the non-covalent interactions (NCI) with the NCI method (Johnson et al., 2010), which considers the distribution of the electron density (ρ), its gradient (s) and its second derivatives matrix (λ_1_, λ_2_, λ_3_). A comprehensive picture can be drawn using different plots of these quantities. According to the color code reported on the graphics, the isosurfaces visible in the NCI plots represent the area for attractive and repulsive interactions.

## Results and discussion

### Conformational space

**Figure 1:**
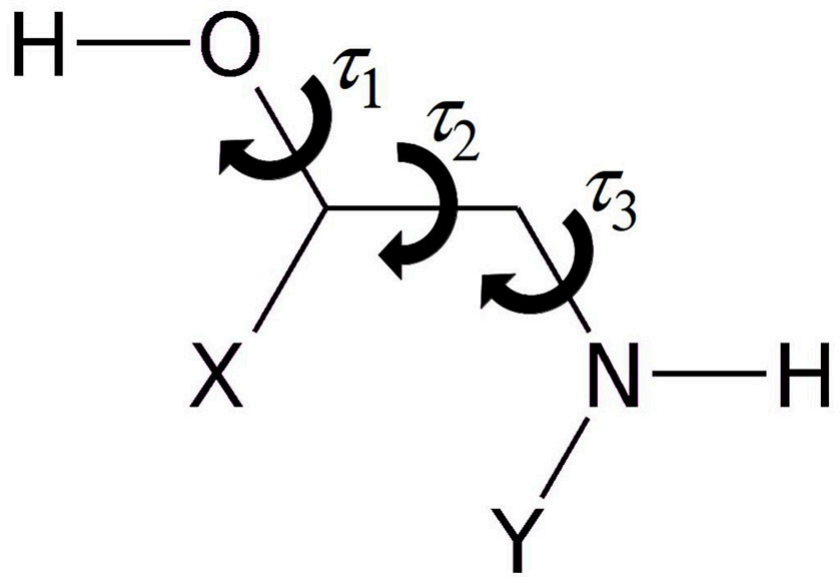
Sketch of 1,2-aminoalcohols (Y = H, CH_3_; X = H, phenyl, catechol) and significant dihedral angles describing the conformational space.

As represented in Figure 1, the rotamers of 1,2-aminoalcohols can be described according to the torsional arrangement of the hydroxyl group (τ_1_ = HOCC), the skeletal frame (τ_2_ = OCCN), and the orientation of the amino group (τ_3_ = CCNY). In general, because of steric hindrance there are three possible staggered configurations for each dihedral angle: *gauche* at τ ≈ 60° (G), *trans* at τ ≈ 180° (T) and *gauche*' at τ ≈ 300° (G'), which lead to 33 = 27 possible rotamers, labeled as τ_1_τ_2_τ_3_, where τ can be *G, T, G'*. In order to facilitate the identification, we use upper case letters for the skeletal and amino orientations and lower case letters for the hydroxyl orientation.

In 1,2-aminoalcohols, both the hydroxyl and the amino groups can act as HB donor or acceptor, but the preference of the amino group to act as HB acceptor in this kind of molecules is well documented by several studies (Penn and Curl, 1971; Penn and Buxton, 1975; Räsänen et al., 1982; Silva et al., 1999; Widicus et al., 2003; Liu et al., 2004; Seurre et al., 2004; Asselin et al., 2016). For this reason, the most stable conformers are those in which an interaction between the hydroxyl and the amino groups takes place to form an intramolecular HB of the O-H···N type. When the OCCN frame (τ_2_) is in the *trans* configuration, the hydroxyl and amino groups are too far apart to interact with each other. The interaction is also forbidden in the *GG'* and *G'G* type rotamers, but it may take place in the *G'G*', *GG, GT, G'T* forms. In particular, the relative orientation of the groups allows for a NH···O interaction in the *G'G'* and *GT* forms and for a OH···N interaction in the *GG* and *G'T* conformers.

**Figure 2:**
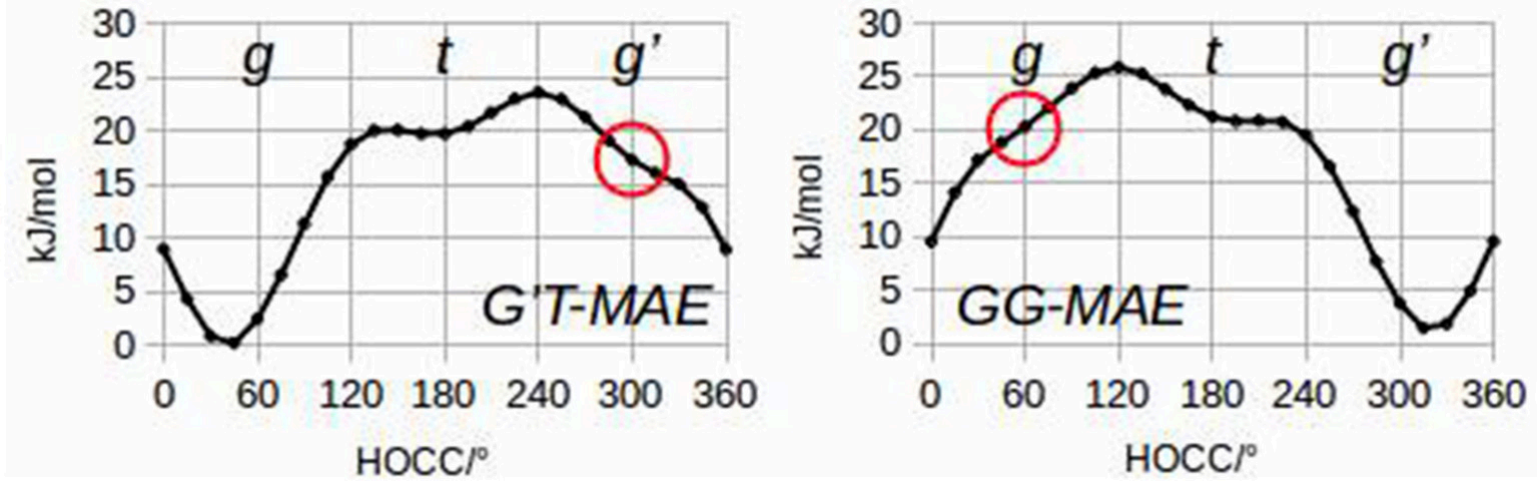
Hydroxyl internal rotation pathways of *G'T*-MAE and *GG*-MAE.

To quantify these considerations, free geometry optimizations of MAE were run for each of its 27 rotamers. It has been possible to localize 24 minima at the B3LYP/6-311++G(d,p) level of calculation, and 23 minima using MP2/6-311++G(d,p). Subsequent analysis of the hydroxyl internal rotation pathway (executed at the B3LYP/6-311++G(d,p) level and performed changing τ_1_ by a 15° step while all other coordinates were freely optimized), clearly shows that rotamers such as *g'G'T* and *gGG* do not represent minima on the PES (see Figure 2).

**Figure 3:**
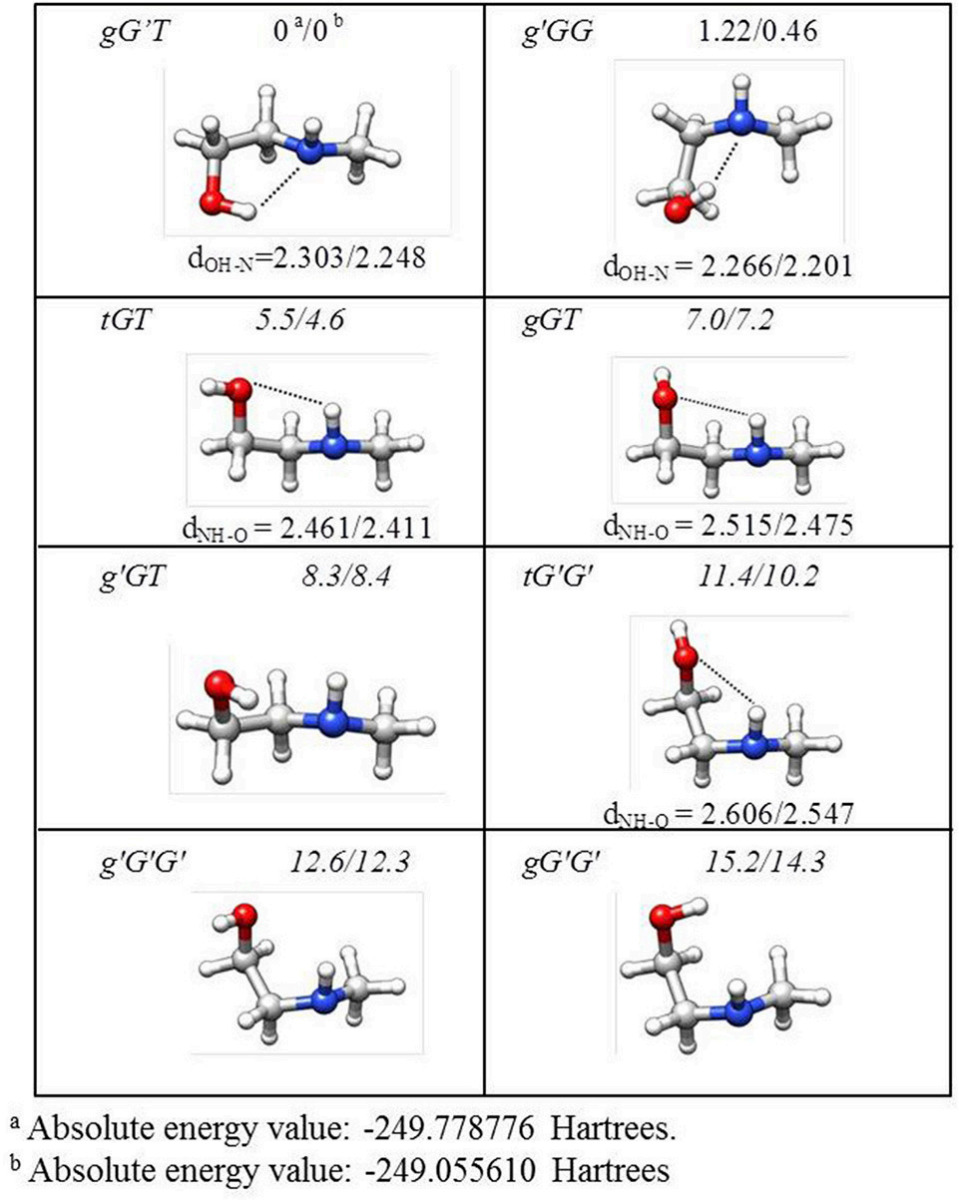
Theoretical shapes, electronic relative energies values (kJ mol^−1^), and HB distances (Å) for the most stable conformers of MAE. First and second values refer to B3LYP/6-311++G(d,p) and MP2/6-311++G(d,p) results, respectively.

The lower energy conformers are shown in Figure 3 with their relative energy values. The *gG'T* conformer represents the global minimum characterized by an intramolecular OH···N HB, followed by the *g'GG*, that shows the same kind of intramolecular interaction, and lies only about 1 kJ mol^−1^ above. Then two conformers exhibiting an intramolecular NH···O HB lie at about 5 (*tGT*) and 7 (*gGT*) kJ mol^−1^. Four other conformations lie between 8 and 15 kJ mol^−1^, while the remaining ones above 18 kJ mol^−1^.

Given the calculated stabilization energies and the values of the dipole moment components, we estimated that only the two most stable conformations, *gG'T* and *g'GG*, would be sufficiently populated in the free jet expansion to allow a spectroscopic detection, thus the analysis was centered on them. Table 1 reports their calculated spectroscopic parameters: rotational constants (*A, B, C*), quartic centrifugal distortion constants (*D*_J_, *D*_JK_, *D*_K_, *d*_1_, *d*_2_), potential barrier for the methyl internal rotation (*V*_3_) dipole moment components in the principal axes system (μ_a_, μ_b_, μ_c_) and nuclear quadrupole coupling constants related to the ^14^N nucleus (χ_*aa*_, χ_*bb*_, χ_cc_). As for the dipole moment component's signs, this is determined once the choice of axis is made; in this case the axes are chosen so that the C atom linked to the oxygen atom has all positive coordinates.

**Table 1:** Calculated [basis set 6-311++G(d,p)], rotational constants (*A, B, C*), quartic centrifugal distortion constants (*D*_J_, *D*_JK_, *D*_K_, *d*_1_, *d*_2_), potential barrier for the methyl internal rotation (*V*_3_) dipole moment components in the principal axes system (μ_a_, μ_b_, μ_c_), and nuclear quadrupole coupling constants related to the ^14^N nucleus (χ_*aa*_, χ_*bb*_, χ_cc_) of *gG'T* and *g'GG-MAE*.

	***gG'T***		***g'GG***	
	**B3LYP**	**MP2**	**B3LYP**	**MP2**
*A/*MHz	11917	11955	9295	9202
*B/*MHz	2652	2691	3004	3090
*C/*MHz	2394	2431	2807	2890
*D_*J*_*/kHz	1.2	1.17	3.40	4.07
*D_*JK*_/*kHz	−7.0	−8.43	−21.80	−27.07
*D_*K*_/*kHz	66.0	68.25	68.70	78.44
*d_1_ /*kHz	−0.23	−0.22	−0.66	−0.80
*d_2_/*kHz	−0.01	−0.01	−0.01	−0.02
*V_3_/*kJ mol^−1^	11.8	12.7	9.8	10.7
*μ_*a*_*/D	2.48	2.53	−2.80	−2.86
*μ_*b*_*/D	1.46	1.60	1.34	1.44
*μ_*c*_*/D	0.87	0.87	0.09	0.23
*μ_*tot*_*/D	3.00	3.11	3.11	3.21
*χ_*aa*_/*MHz	1.62	1.49	0.56	0.53
*χ_*bb*_/*MHz	−3.86	−3.57	−2.75	−2.32
*χ_*cc*_/*MHz	2.25	2.08	2.19	1.80
Δ*E*_e_/kJ mol^−1^	0^a^	0^b^	1.22	0.46
Δ*E*_0_/kJ mol^−1^	0^c^	0^d^	1.32	0.65

a *Absolute energy value: −249.778776 Hartrees*.

b *Absolute energy value: −249.055610 Hartrees*.

c *Absolute energy value: −249.652558 Hartrees*.

d *Absolute energy value: −248.926849 Hartrees*.

### MAE rotational spectrum

**Figure 4:**
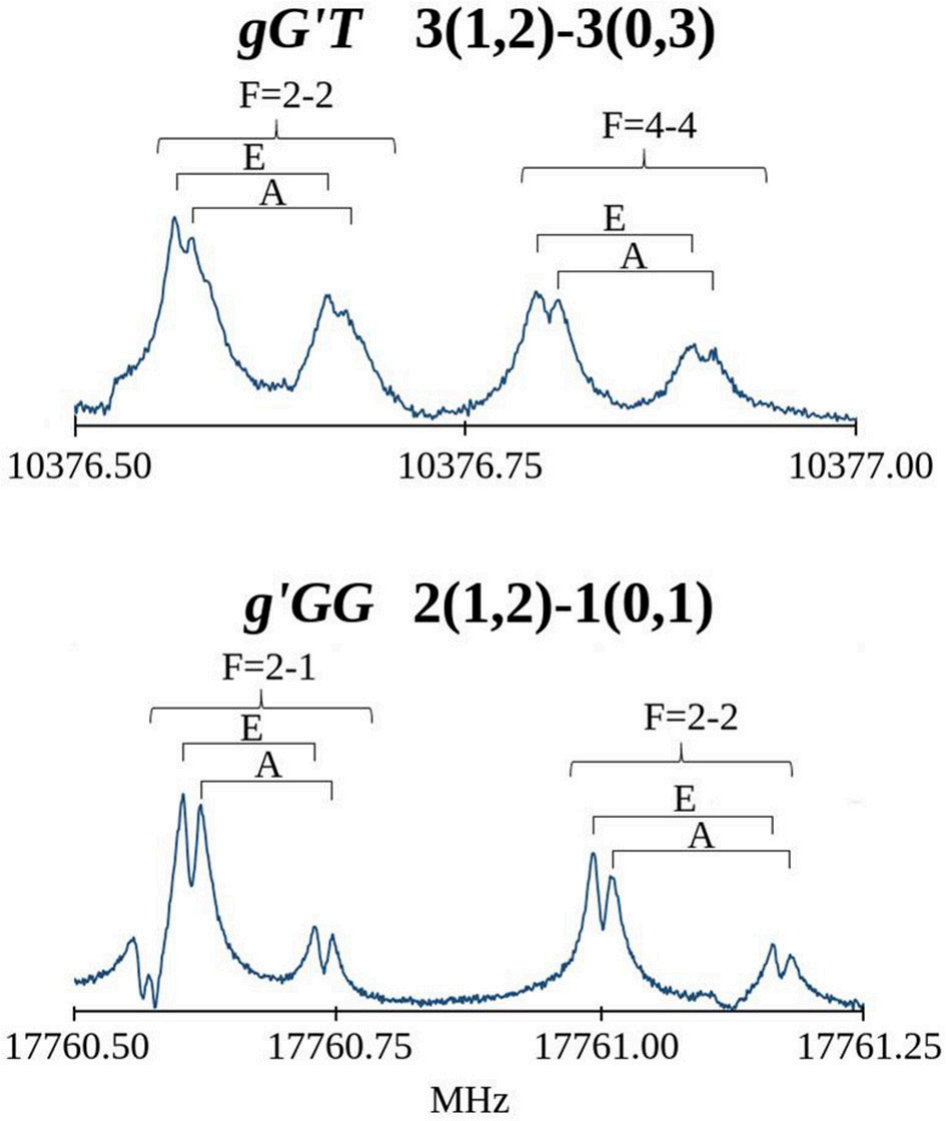
Recorded transitions of MAE showing the splitting of the rotational transitions due to the ^14^N nuclear quadrupole coupling (*F* quantum number) and the methyl group internal rotation (A/E).

The first spectral scans were recorded with the FJAMMW spectrometer in the 59.6-74.4 GHz frequency range and the transitions were assigned relying on the predictions based on previous work (Penn and Buxton, 1975) and supported by our quantum chemical calculations. The rotational spectra showed μ_a_-, μ_b_- and μ_c_-type *R*-branch transitions and for the *g'GG* conformer also few μ_b_- and μ_c_- type *Q*-branch transitions. Some of these lines also presented a hyperfine ^14^N quadrupole structure while the torsional splitting due to the methyl internal rotation was not measurable in this frequency range. Both splittings were indeed very clearly resolved in the spectrum recorded with the cavity-based FTMW spectrometer, as can be seen in Figure 4. The overall fittings for both conformers, including the rotational lines from both frequency ranges, were carried out using the XIAM program (Hartwig and Dreizler, 1996), obtaining the parameters reported in Table 2. The rotational lines reported previously were not included in the fit due to the low accuracy with which they were measured. The frequency range covered by this experiment warrants the determination of all significant constants. All the measured frequencies are listed in Table S1 for the *gG'T* conformer and in Table S9 for the *g'GG* one.

**Table 2:** Experimental rotational constants (*A, B, C*), quartic centrifugal distortion constants (*D*_J_, *D*_JK_, *D*_K_, *d*_1_, *d*_2_), potential barrier (*V*_3_), and structural parameters (*F*_0_, δ, ε) for the methyl internal rotation and the nuclear quadrupole coupling constants related to the ^14^N nucleus (χ_*aa*_, χ_*bb*_, χ_cc_) of *gG'T*-MAE and *g'GG*-MAE.

	***gG'T***	***g'GG***
*A*/MHz	12123.7430(6)^a^	9155.314(2)
*B*/MHz	2653.8058(2)	3076.3199(5)
*C*/MHz	2400.7362(2)	2868.6381(4)
*D_*J*_*/kHz	1.1589(7)	4.341(4)
*D_*JK*_*/kHz	−8.389(6)	−28.83(2)
*D_*K*_*/kHz	70.88(8)	84.10(7)
*d_1_*/kHz	−0.2207(5)	−0.927(3)
*d_2_*/kHz	−0.0107(5)	−0.001(2)
*χ_*aa*_*/MHz	1.578(2)	0.510(7)
*(χ_*bb*_* – *χ_*cc*_*)/MHz	−5.262(3)	−3.81(1)
*V_3_/*kJ mol^−1^	11.91(8)	9.7(1)
*F*_0_/GHz	157.09638^b^	157.24301^b^
*δ/*deg	2.7598^b^	0.8741^b^
*ε/d*eg	1.4853^b^	−1.1993^b^
σ^c^/MHz	0.006	0.02
*N*^d^	206	202

a *Error in parentheses in units of the last digit*.

b *Fixed to the MP2/6-311++G(d,p) calculated value*.

c *Root-mean-square deviation of the fit*.

d *Number of lines in the fit*.

The high sensitivity and resolution of the FTMW technique allows also for the observation of isotopically substituted species in natural abundance. For MAE, the spectra of all ^13^C and ^15^N species of both conformers were measured. More data can be obtained using hydroxyl and amino enriched deuterated species and this analysis was performed with the FJ-AMMW spectrometer for the global minimum *gG'T*. The analysis of the rotational transitions was carried out in the same way described before, and all frequencies and spectroscopic parameters for the isotopologues are reported in Tables S2–S8, S14 for the *gG'T* conformer and Tables S10–S13, S15 for the *g'GG* one. The isotopic study performed, allowed to increase the accuracy regarding the structural determination of MAE.

### MAE structure

**Figure 5:**
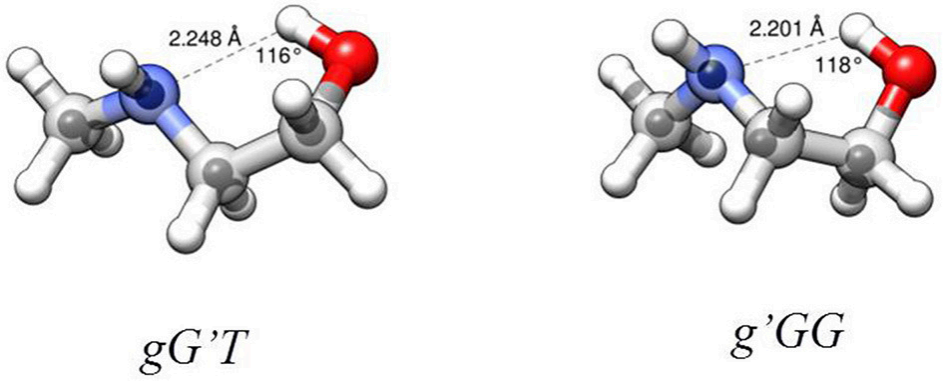
Structures of the observed two most stable conformers of MAE. Larger circles are from the B3LYP/6-311++G(d,p) optimizations, small circles represent the position of the substituted atoms in the *r*_s_ structure. HB parameters from *r*_0_ structure.

From the experimental rotational constants obtained for the different isotopic species (labeling of atoms in Figure S1), the experimental *r*_s_-structure using Kraitchman's substitution method (Kraitchman, 1953) was determined for the observed conformers of MAE. This analysis leads to the determination of the principal axis coordinates of the substituted atom from the changes in the principal moments of inertia resulting from a single isotopic substitution. Those values are reported in Tables S16, S18 and compared with the coordinates obtained from the theoretical calculations (reported in Tables S20–S27). A partial structural *r*_0_ fit aimed at reproducing the experimental rotational constants starting from the calculated geometry (MP2) and involving τ_3_ and all the heavy atom valence angles, was also performed with the STRFIT program (Kisiel, 2001). The structure obtained from the *r*_0_ fit is reported in the Tables S17, S19, while a representation of the structural results with the derived HB lengths for both conformers is shown in Figure 5. From the data reported in the tables and visualized in the figure, the good accuracy of the structural predictions can be asserted. In both species, and especially in the *g'GG* conformer the position of the ethylenic carbon atom closest to the amino group (C3) seems to be not so accurately predicted. This could be a limitation of the substitution method as this atom is the closest to the origin of the inertial axes system and in the case of the *g'GG* conformer to the lower accuracy with which the rotational constants were determined. The derived HB lengths show that the less stable conformer has the shorter one. Since shorter bond length should be related to strongest bonds, this result seems counterintuitive, so other factors must influence the relative stability between conformers (see next section). Looking at the values of *A, B* and *C*, which reflect the structural distribution about the rotational axes, MP2 appears to be the method that better reproduces the experimental data (obs-calc^MP2^ <1.5%) with respect to B3LYP (1.5% < obs-calc^B3LYP^ <3%). This comparison between the experimental and theoretical rotational constants shows that also using a relatively extended basis set (6-311++G(d,p)), the discrepancies between calculated and experimental rotational constants of this small system, characterized by various degrees of freedom, is greater with respect that of other more rigid ones (obs-calc^MP2^ <0.4%) (Melandri et al., 2010a). The accuracy of molecular structures and energies obtained with different theoretical methods and the relation of the accuracy to the computational cost is highly debated in the literature. A thorough discussion is beyond the scope of this work as papers can be found which are devoted entirely to these kind of studies (see for example Grimme and Steinmetz, 2013).

Although it has been shown that DFT methods are less accurate than for weakly bound systems (Bartolomei et al., 2011, 2014), in this particular case, and concerning the relative energies between the *gG'T* and *gG'G* conformers, we can state that the B3LYP calculation (Δ*E*_e_ = 1.22 kJ mol^−1^; Δ*E*_0_ = 1.32 kJ mol^−1^) better reproduces the experimentally determined value of 1.9 (3) kJ mol^−1^ (Penn and Curl, 1971) with respect to the MP2 one (Δ*E*_e_ = 0.46 kJ mol^−1^; Δ*E*_0_ = 0.65 kJ mol^−1^). The same is true also for the methyl internal rotation barrier: the experimental value for the *gG'T* and *g'GG* conformers are 11.9 and 9.7 kJ mol^−1^ respectively, while the two values [B3LYP and MP2/6-311++G(d,p)] are 11.8/12.7 and 9.8/10.7 kJ mol^−1^. As regards the electric dipole moment, the experimental value [2.86 (1) D] (Penn and Buxton, 1975) is closer to the B3LYP result (3.00 D) than to the MP2 (3.11 D) one.

### Non-covalent interactions and adrenergic neurotransmitters

Because of its ability to reproduce the molecular electronic properties of MAE and also for its convenient computational cost, the B3LYP/6-311++G(d,p) method was selected to explore the conformational space of a series of analogs of the adrenergic neurotransmitters containing the 1,2-aminoalcohol frame. In particular three aminoalcohols (AE, APE, and noradrenaline) and three *N*-methylamino-alcohols (MAE, MAPE and adrenaline) were considered, in order to compare the changes and similarities in their conformational properties by substitution of atoms or functional groups. The analysis is restricted to the lower energy conformers, stabilized by the intermolecular OH···N HB, and when different enantiomers are possible, only the (R)-form is considered.

**Figure 6:**
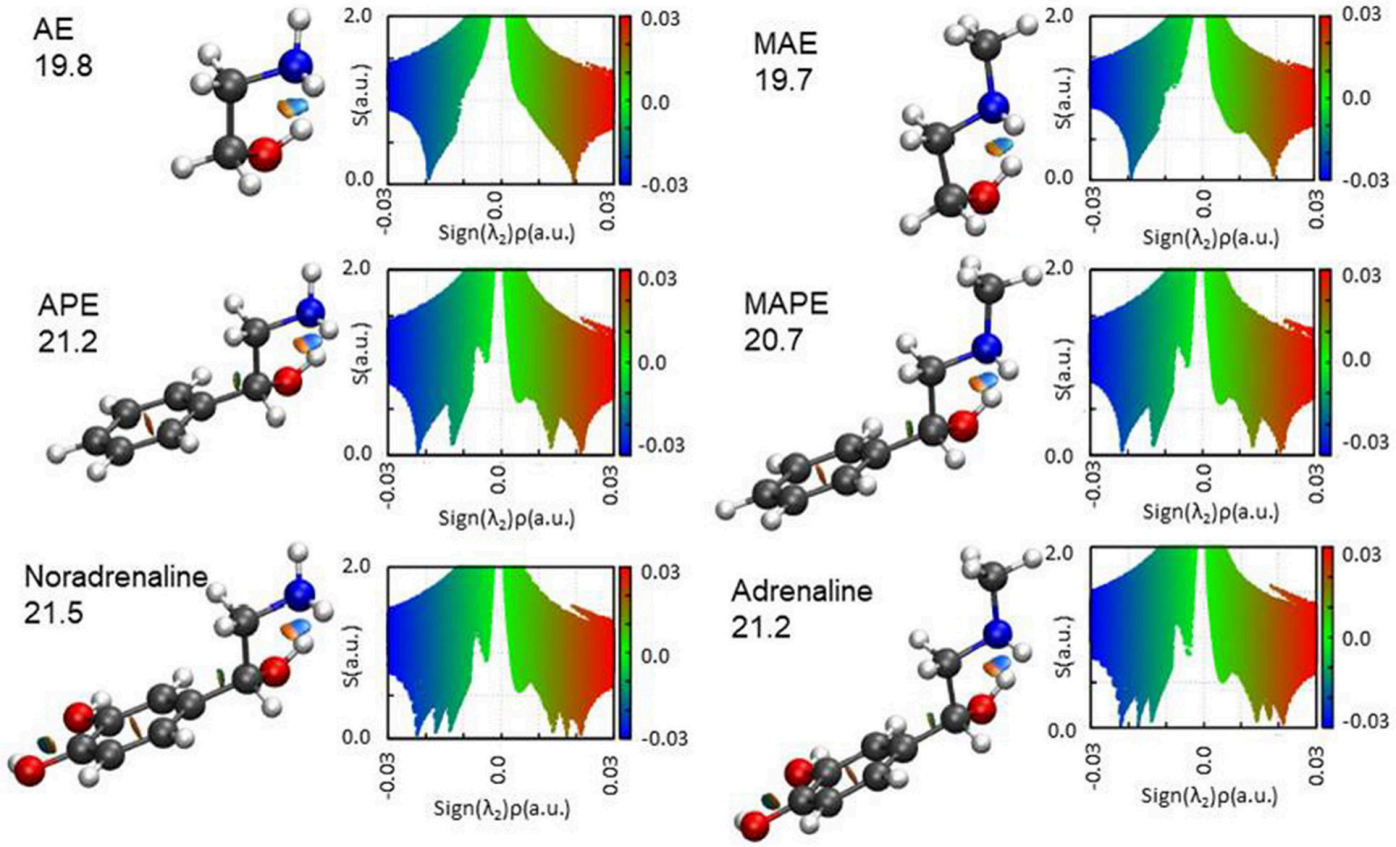
The NCI plots and AIM binding energies (OH···N HB in kJ mol^−1^) for the studied molecules. Blue and green colors identify the presence of strong and weak attractive interactions, respectively. Red color indicates repulsive interaction.

The results of the NCI representation are reported in Figure 6. For all molecules, the main interaction (an OH···N HB) is clearly visible as a colored region between the two atoms involved. The diagrams show that this interaction (that with the largest value of ρ) has a mixed attractive-repulsive character shown by the presence of two point where *s* = 0 and λ_2_ positive (repulsive interaction) or negative (attractive interaction). From the comparison among the different molecular systems, it can be seen that no substantial change in the nature of the interaction arises attaching an aromatic ring or a catechol ring to the 1,2-aminoalcohol frame. A deeper analysis of the electronic density gradient can be done on the diagrams reported for each molecule and the comparison of the AIM calculated binding energies (also indicated in the figure for each molecule). These data show that the HB binding energies (all calculated to be around 20 kJ mol^−1^, see Figure 6) are not affected when the methyl group substitutes an hydrogen atom of the amino group (that is in going from AE to MAE, from APE to MAPE or from noradrenaline to adrenaline), while an increment can be observed with substitution by a benzene or even more by catechol ring.

The AIM binding energies for different conformations of the molecules show that the value is greater when the methyl group is in the *gauche* position (21.2 kJ mol^−1^) with respect to the *trans* (19.7 kJ mol^−1^) while the stability is reversed, that is: *gG'T* is the global minimum while *g'GG* is slightly higher in energy. One could question the accuracy of the AIM method in estimating the intramolecular binding energies, but an independent piece of evidence can be found in the values of the OH···N intramolecular bond. The theoretical values and their trends (B3LYP/and MP2/6-311++G(d,p)) for *gG'T* (d_OH−N_ = 2.303/2.248 Å) and *g'GG* (d_OH−N_ = 2.266/2.201 Å) are in agreement with the experimental ones (d_OH−N_ = 2.271 Å for *gG'T* and d_OH−N_ = 2.233 Å for *g'GG*) validating the theoretical calculations. All these results are in agreement with the idea that the HB length is indeed shorter when the binding energy is higher indicating that for these molecular systems the strength of the HB is not the only factor influencing the stability of the conformers.

**Figure 7:**
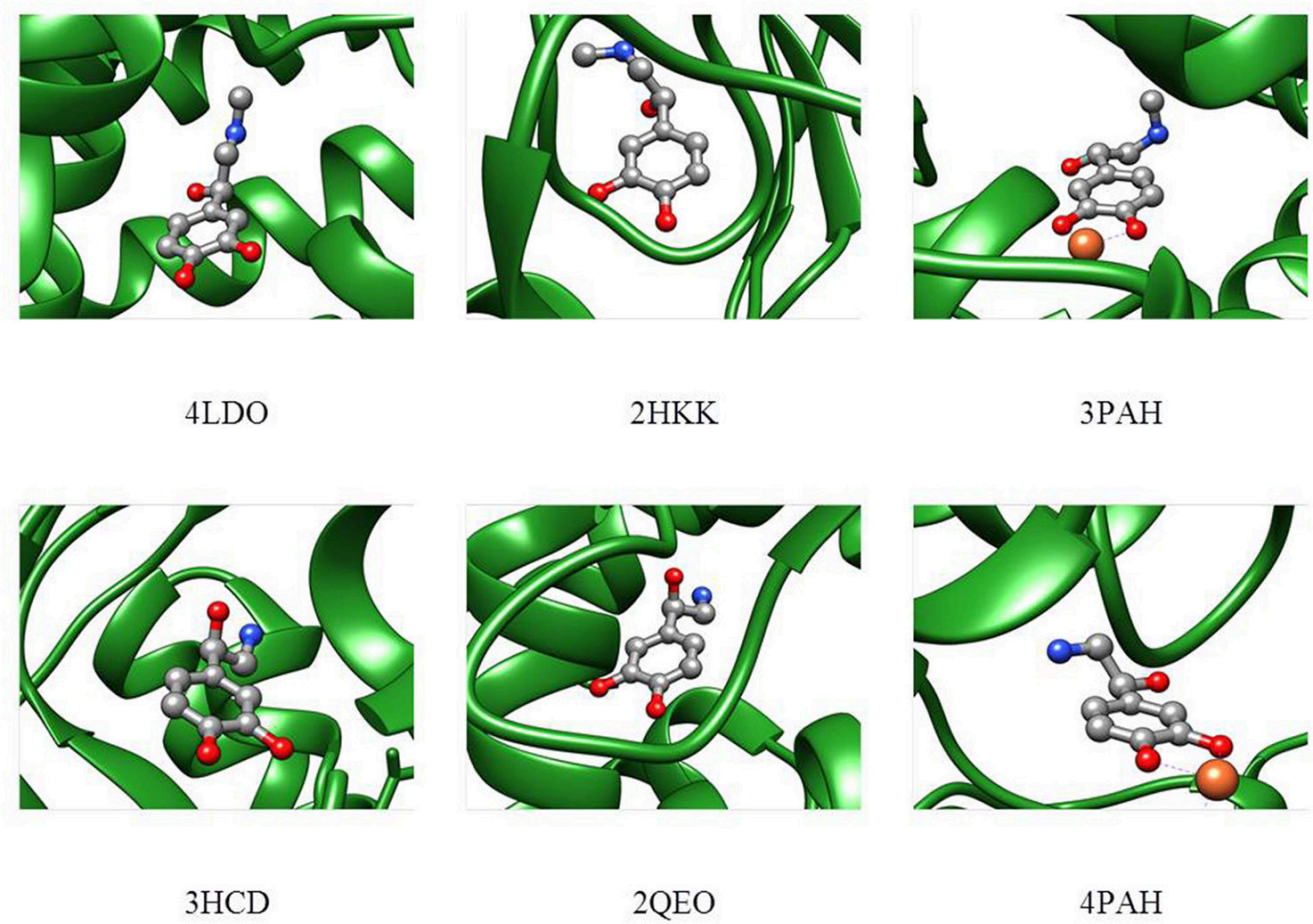
Adrenaline (upper part) and noradrenaline (lower part) shapes in the receptor as deduced from the Protein Data Bank files.

In order to compare the conformations studied in the isolated phase to those adopted in the biological environment, a search for the adrenaline and noradrenaline ligands was carried out on the Worldwide Protein Data Bank (Berman et al., 2003). The analyzed structures for adrenaline [4LDO (Ring et al., 2013), 2HKK (Temperini et al., 2007), 3PAH (Erlandsen et al., 1998)] and noradrenaline [3HCD (Drinkwater et al., 2009), 2QEO (Mans et al., 2007), 4PAH (Erlandsen et al., 1998)] present the biomolecule in different conformations that cannot be easily superimposed to the calculated structural minima of the isolated phase. Nevertheless, it can be seen in Figure 7 that, even if other interactions with the receptor are present, in each of the structures, the relative position of the amino and alcoholic group is such that they could still interact through an intramolecular HB (4LDO, 2HKK, 3HCD, 2QEO). When the neurotransmitter is involved in an interaction with an iron ion (3PAH and 4PAH), the two functional groups are too far apart to interact.

## Conclusions

The combined experimental and theoretical study of the conformational space of the small and flexible MAE molecule studied in the isolated phase, allowed the understanding of the driving forces and factors influencing its stability. The analysis performed in the isolated phase shows that the two lowest energy conformations are strongly stabilized by an intermolecular OH···N HB that was structurally characterized and energetically quantified. The straightforward comparison between the experimental (11 isotopologues measured) and calculated structures assesses that the accuracy of both MP2 and B3LYP theoretical methods in reproducing the structural parameters, although generally good, is slightly less than that obtained for more rigid molecular systems. This evidences that the isolated phase experiments and the derived data, are important to benchmark the calculations. The B3LYP method seems superior in estimating the relative energies of the conformations and the methyl group internal rotation barriers, and was thus used for the study of other 1,2-aminoalcohols including adrenaline and noradrenaline and their molecular models APE and MAPE. For all molecules, the lowest energy conformation is shown to be characterized by the same HB interaction shown by MAE. The energy of the HB has been calculated for all molecular systems and it increases when the benzene ring (APE, MAPE), and even more the catechol ring (noradrenaline, adrenaline) is present. The presence or absence of the methyl group attached to the aminic function seems not to affect the subtle balance of forces characterizing the conformational space of this kind of molecules.

## Author contributions

CC, AM, and SM conceived the experiment. All authors conducted the experiments and analyzed the results. SM wrote the manuscript and all authors reviewed the manuscript.

### Conflict of interest statement

The authors declare that the research was conducted in the absence of any commercial or financial relationships that could be construed as a potential conflict of interest.
