# Supplementary material for: Rotational Spectrum and Conformational Analysis of N-Methyl-2-Aminoethanol: Insights into the Shape of Adrenergic Neurotransmitters
Source: Front Chem. 2018 Feb 22;6:25. doi: 10.3389/fchem.2018.00025 (PMC5827360; doi:10.3389/fchem.2018.00025)
Supplement: Supplementary file 1 [file Table1.DOCX]

**Rotational Spectrum and Conformational Analysis of *N*-methyl-2-aminoethanol: Insights into the Shape of Adrenergic Neurotransmitters**

Camilla Calabrese,^1,2^ Assimo Maris,^1^ Luca Evangelisti,^1^ Anna Piras,^1^ Valentina Parravicini,^1^ and Sonia Melandr^1,^*

^1^Dipartimento di Chimica “G. Ciamician” dell’Università, via Selmi 2, I-40126 Bologna, Italy

^2^Present address: Dpto. Química Física, Facultad de Ciencia y Tecnología Universidad del País Vasco (UPV/EHU), Apartado 644, E-48080 Bilbao, Spain.

*Corresponding author: Sonia Melandri

E-mail address: sonia.melandri@unibo.it

Figure S1. Numbering of atoms in MAE

Table S1. Experimental transition frequencies of MAE conformer *gG’T*

Table S2. Experimental transition frequencies of MAE conformer *gG’T* isotopologue ^13^C_1

Table S3. Experimental transition frequencies of MAE conformer *gG’T* isotopologue ^13^C_3

Table S4. Experimental transition frequencies of MAE conformer *gG’T* isotopologue ^13^C_4

Table S5. Experimental transition frequencies of MAE conformer *gG’T* isotopologue ^15^N

Table S6. Experimental transition frequencies of MAE conformer *gG’T* isotopologue CH(OD)CHN(D)CH_3_

Table S7. Experimental transition frequencies of MAE conformer *gG’T* isotopologue CH(OH)CHN(D)CH_3_

Table S8. Experimental transition frequencies of MAE conformer *gG’T* isotopologue CH(OD)CHN(H)CH_3_

Table S9. Experimental transition frequencies of MAE conformer *g’GG*

Table S10. Experimental transition frequencies of MAE conformer *g’GG* isotopologue ^13^C_1

Table S11. Experimental transition frequencies of MAE conformer *g’GG* isotopologue ^13^C_3

Table S12. Experimental transition frequencies of MAE conformer *g’GG* isotopologue ^13^C_4

Table S13. Experimental transition frequencies (MHz) of MAE conformer *g’GG* isotopologue ^15^N

Table S14. Spectroscopic constants of MAE conformer *gG’T* isotopologues

Table S15. Spectroscopic constants of MAE conformer *g’GG* isotopologues

Table S16 Principal axis coordinates of heavy atoms in MAE *gG’T*

Table S17 Partial r_0_ structure of MAE *gG’T*. Calculated geometry (MP2/6-311++G**) and fitted parameters (in bold)

Table S18 Principal axis coordinates of heavy atoms in MAE *g’GG*

Table S19 Partial r_0_ structure of MAE *g’GG*. Calculated geometry (MP2/6-311++G**) and fitted parameters (in bold)

Table S20. MP2/6-311**G(p,d) structure of the *g'GG* MAE conformer.

Table S21. MP2/6-311**G(p,d) structure of the *gG'G'* MAE conformer.

Table S22. MP2/6-311**G(p,d) structure of the *g'GT* MAE conformer.

Table S23. MP2 6-311**G(p,d) structure of the *tGT* MAE conformer.

Table S24. MP2 6-311**G(p,d) structure of the *gGT* MAE conformer.

Table S25. MP2 6-311**G(p,d) structure of the *gG'G'* MAE conformer.

Table S26. MP2 6-311**G(p,d) structure of the *tG'G'* MAE conformer.

Table27. MP2 6-311**G(p,d) structure of the *g'G'G'* MAE conformer.


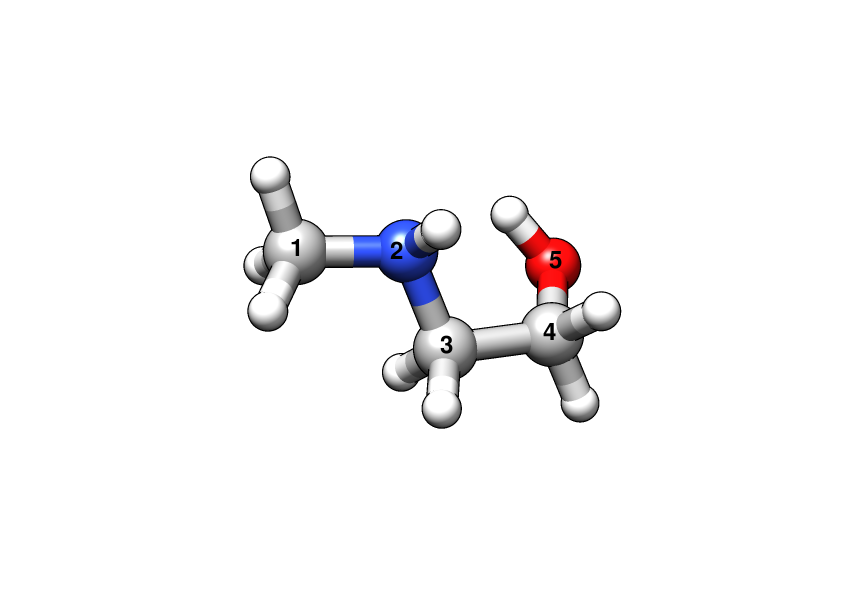


Figure S1. Numbering of atoms in MAE

Table S1. Experimental transition frequencies of MAE conformer *gG’T*

| *J’_K’a_,_K’c_* | *2F’* | *J”_K"a_,_K”c_* | *2F”* |  | *ν*_OBS_/MHz | ∆*ν*_OBS-CALC_/MHz |
| --- | --- | --- | --- | --- | --- | --- |
| 2_0,2_ | 2 | 1_0,1_ | 2 |  | 10103.2772 | 0.0033 |
|  | 6 |  | 4 |  | 10104.0163 | -0.0001 |
|  | 4 |  | 2 |  | 10104.0274 | -0.0004 |
|  | 2 |  | 0 |  | 10104.4612 | 0.0041 |
|  | 4 |  | 4 |  | 10104.5044 | 0.0033 |
| 2_1,1_ | 2 | 1_1,0_ | 2 |  | 10360.7644 | 0.0061 |
|  | 6 |  | 4 |  | 10361.9212 | -0.0002 |
|  | 2 |  | 0 |  | 10362.1398 | 0.0002 |
|  | 4 |  | 2 |  | 10362.4738 | 0.0059 |
|  | 4 |  | 4 |  | 10363.0237 | 0.0033 |
| 2_1,2_ | 4 | 1_1,1_ | 4 |  | 9855.4582 | 0.0019 |
|  | 6 |  | 4 |  | 9856.0474 | -0.0008 |
|  | 4 |  | 2 |  | 9856.4829 | 0.0009 |
| 3_0,3_ | 4 | 2_0,2_ | 4 |  | 15142.8488 | -0.0016 |
|  | 6 |  | 4 |  | 15143.4783 | -0.0036 |
|  | 8 |  | 6 |  | 15143.4997 | 0.0009 |
|  | 4 |  | 2 |  | 15143.5970 | -0.0073 |
|  | 6 |  | 6 |  | 15143.9663 | -0.0003 |
| 3_1,2_ | 6 | 2_1,1_ | 6 |  | 15541.0572 | -0.0049 |
|  | 4 |  | 2 |  | 15539.9617 | -0.0047 |
|  | 6 |  | 4 |  | 15539.9617 | -0.0013 |
|  | 8 |  | 6 |  | 15539.7972 | -0.0010 |
|  | 4 |  | 4 |  | 15538.2528 | -0.0040 |
| 3_1,3_ | 6 | 2_1,2_ | 6 |  | 14780.4792 | -0.0014 |
|  | 4 |  | 2 |  | 14780.8141 | 0.0014 |
|  | 8 |  | 6 |  | 14780.9703 | 0.0001 |
|  | 6 |  | 4 |  | 14781.0721 | -0.0005 |
|  | 4 |  | 4 |  | 14781.7313 | -0.0022 |
| 1_1,1_ | 2 | 0_0,0_ | 2 | E | 14523.5967 | 0.0003 |
|  |  |  |  | A | 14523.6119 | 0.0010 |
|  | 4 |  | 2 | E | 14524.6234 | 0.0012 |
|  |  |  |  | A | 14524.6367 | 0.0000 |
|  | 0 |  | 2 | E | 14526.1591 | -0.0018 |
|  |  |  |  | A | 14526.1745 | -0.0009 |
| 1_1,0_ | 0 | 0_0,0_ | 2 | E | 14776.5273 | -0.0015 |
|  |  |  |  | A | 14776.5452 | 0.0021 |
|  | 4 |  | 2 | E | 14777.3595 | 0.0019 |
|  |  |  |  | A | 14777.3732 | 0.0013 |
|  | 2 |  | 2 | E | 14777.9159 | 0.0058 |
|  |  |  |  | A | 14777.9304 | 0.0060 |
| 1_1,0_ | 0 | 1_0,1_ | 2 | E | 9721.5986 | 0.0015 |
|  |  |  |  | A | 9721.6157 | 0.0044 |
|  | 4 |  | 2 | E | 9722.4264 | 0.0006 |
|  |  |  |  | A | 9722.4417 | 0.0016 |
|  | 4 |  | 4 | E | 9722.8999 | 0.0008 |
|  |  |  |  | A | 9722.9148 | 0.0015 |
|  | 2 |  | 2 | E | 9722.9838 | 0.0055 |
|  |  |  |  | A | 9722.9838 | -0.0088 |
|  | 2 |  | 4 | E | 9723.4459 | -0.0057 |
|  |  |  |  | A | 9723.4608 | -0.0050 |
|  | 2 |  | 0 | E | 9724.1680 | 0.0065 |
|  |  |  |  | A | 9724.1822 | 0.0065 |
| 2_1,1_ | 2 | 2_0,2_ | 4 | E | 9979.7066 | -0.0023 |
|  |  |  |  | A | 9979.7215 | -0.0016 |
|  | 6 |  | 4 | E | 9980.3191 | -0.0004 |
|  |  |  |  | A | 9980.3345 | 0.0008 |
|  | 2 |  | 2 | E | 9980.4551 | -0.0077 |
|  |  |  |  | A | 9980.4688 | -0.0082 |
|  | 6 |  | 6 | E | 9980.8051 | 0.0010 |
|  |  |  |  | A | 9980.8161 | -0.0022 |
|  | 4 |  | 4 | E | 9981.4155 | -0.0030 |
|  |  |  |  | A | 9981.4307 | -0.0020 |
|  | 4 |  | 6 | E | 9981.9032 | 0.0000 |
|  |  |  |  | A | 9981.9134 | -0.0040 |
|  | 4 |  | 2 | E | 9982.1657 | -0.0068 |
|  |  |  |  | A | 9982.1798 | -0.0068 |
| 3_1,2_ | 4 | 3_0,3_ | 6 | E | 10376.1910 | -0.0026 |
|  |  |  |  | A | 10376.2090 | 0.0014 |
|  | 4 |  | 4 | E | 10376.8212 | -0.0039 |
|  |  |  |  | A | 10376.8345 | -0.0046 |
|  | 8 |  | 8 | E | 10377.1078 | 0.0041 |
|  |  |  |  | A | 10377.1206 | 0.0029 |
|  | 6 |  | 6 | E | 10377.8996 | -0.0002 |
|  |  |  |  | A | 10377.9140 | 0.0002 |
|  | 6 |  | 4 | E | 10378.5286 | -0.0027 |
|  |  |  |  | A | 10378.5467 | 0.0014 |
| 4_1,3_ | 6 | 4_0,4_ | 6 | E | 10922.5412 | -0.0020 |
|  |  |  |  | A | 10922.5557 | -0.0012 |
|  | 10 |  | 10 | E | 10922.7754 | -0.0001 |
|  |  |  |  | A | 10922.7937 | 0.0044 |
|  | 8 |  | 8 | E | 10923.6821 | 0.0031 |
|  |  |  |  | A | 10923.6971 | 0.0043 |
| 5_1,4_ | 8 | 5_0,5_ | 8 | E | 11631.9011 | -0.0024 |
|  |  |  |  | A | 11631.9116 | -0.0053 |
|  | 12 |  | 12 | E | 11632.1075 | 0.0026 |
|  |  |  |  | A | 11632.1215 | 0.0033 |
|  | 10 |  | 10 | E | 11633.0929 | -0.0004 |
|  |  |  |  | A | 11633.1121 | 0.0054 |
| 3_2,2_ | 4 | 2_2,1_ | 2 | E | 15163.3220 | 0.0057 |
|  | 8 |  | 6 | A | 15163.5988 | 0.0081 |
|  |  |  |  | E | 15163.5988 | 0.0008 |
|  | 6 |  | 4 | A | 15164.1029 | 0.0051 |
|  |  |  |  | E | 15164.1029 | -0.0022 |
| 3_2,1_ | 4 | 2_2,0_ | 2 | E | 15183.2780 | 0.0033 |
|  |  |  |  | A | 15183.2780 | -0.0045 |
|  | 6 |  | 4 | E | 15184.1111 | 0.0041 |
|  |  |  |  | A | 15184.1111 | -0.0037 |
| 5_0,5_ | 12 | 4_1,4_ | 10 | A | 16778.8358 | -0.0046 |
|  |  |  |  | E | 16778.8492 | -0.0052 |
| 3_3, 0_ |  | 2_2, 0_ |  |  | 63140.24 | 0.0792 |
| 3_3, 0_ |  | 2_2, 1_ |  |  | 63145.11 | -0.0521 |
| 3_3, 1_ |  | 2_2, 1_ |  |  | 63145.11 | 0.0097 |
| 5_3, 2_ |  | 4_2, 3_ |  |  | 73286.68 | 0.0763 |
| 6_2, 4_ | 14 | 5_1, 5_ | 12 |  | 61356.55 | -0.0355 |
|  | 12 |  | 10 |  | 61357.69 | 0.0445 |
| 6_2, 5_ | 14 | 5_1, 5_ | 12 |  | 61009.56 | 0.0281 |
|  | 12 |  | 10 |  | 61010.40 | 0.0482 |
| 7_2, 6_ | 12 | 6_1, 5_ | 10 |  | 61515.72 | -0.0412 |
|  | 14 |  | 12 |  | 61514.66 | 0.0553 |
| 7_2, 5_ | 16 | 6_1, 5_ | 14 |  | 62136.26 | -0.0038 |
|  | 14 |  | 12 |  | 62135.60 | -0.0054 |
| 7_2, 5_ | 16 | 6_1, 6_ | 14 |  | 67442.76 | -0.0193 |
|  | 14 |  | 12 |  | 67443.97 | 0.0132 |
| 7_2, 6_ | 12 | 6_1, 6_ | 10 |  | 66822.03 | 0.0632 |
|  | 16 |  | 14 |  | 66822.03 | -0.0713 |
|  | 14 |  | 12 |  | 66822.92 | -0.0361 |
| 8_2, 7_ | 18 | 7_1, 7_ | 16 |  | 72762.26 | -0.0175 |
|  | 16 |  | 14 |  | 72763.16 | 0.0015 |
| 8_2, 7_ | 14 | 7_1, 6_ | 12 |  | 65693.27 | -0.0137 |
|  | 16 |  | 14 |  | 65692.21 | 0.0387 |
| 8_2, 6_ | 14 | 7_1, 6_ | 12 |  | 66717.94 | -0.0716 |
|  | 18 |  | 16 |  | 66717.94 | 0.0232 |
|  | 16 |  | 14 |  | 66717.33 | -0.0346 |
| 8_2, 7_ | 18 | 7_1, 7_ | 16 |  | 73787.03 | -0.0275 |
|  | 16 |  | 14 |  | 73788.41 | 0.0581 |
| 9_2, 8_ | 16 | 8_1, 7_ | 14 |  | 69753.85 | -0.0181 |
|  | 18 |  | 16 |  | 69752.83 | 0.0377 |
| 9_2, 7_ | 16 | 8_1, 7_ | 14 |  | 71343.41 | -0.0687 |
|  | 20 |  | 18 |  | 71343.41 | -0.0002 |
|  | 16 |  | 14 |  | 71342.96 | -0.0094 |
| 9_1, 8_ | 20 | 8_0, 8_ | 18 |  | 61386.44 | 0.0112 |
|  | 16 |  | 14 |  | 61387.67 | 0.0462 |
| 10_1, 9_ | 18 | 9_0, 9_ | 16 |  | 68044.67 | 0.0932 |
|  | 22 |  | 20 |  | 68044.67 | -0.0327 |
| 10_1, 9_ | 20 | 9_0, 9_ | 18 |  | 68045.95 | -0.0116 |
| 10_2, 9_ | 18 | 9_1, 8_ | 16 |  | 73702.75 | -0.0458 |
|  | 22 |  | 20 |  | 737027.75 | 0.0632 |
|  | 20 |  | 18 |  | 73701.75 | -0.0030 |
| 12_1, 12_ | 22 | 11_0, 11_ | 20 |  | 62645.68 | 0.0113 |
|  | 26 |  | 24 |  | 62645.68 | 0.0473 |
|  | 24 |  | 22 |  | 62645.24 | 0.0503 |
| 14_0, 14_ |  | 13_1, 13_ |  |  | 66341.39 | -0.0563 |
| 14_0, 14_ |  | 13_0, 13_ |  |  | 71218.33 | -0.0872 |
| 15_0, 15_ |  | 14_0, 14_ |  |  | 73792.96 | 0.0594 |
| 15_0, 15_ |  | 14_1, 14_ |  |  | 71590.24 | 0.0128 |
| 15_1, 15_ |  | 14_1, 14_ |  |  | 73386.40 | 0.0465 |
| 15_1, 14_ | 28 | 14_2, 13_ | 26 |  | 60099.09 | 0.0648 |
|  | 32 |  | 30 |  | 60099.09 | 0.0190 |
|  | 30 |  | 28 |  | 60099.81 | 0.0292 |
| 12_1, 11_ |  | 11_1, 10_ |  |  | 61750.98 | -0.0662 |
| 12_2, 11_ |  | 11_2, 10_ |  |  | 60424.22 | -0.0702 |
| 12_2, 10_ |  | 11_2, 9_ |  |  | 61631.84 | -0.0303 |
| 12_3, 10_ |  | 11_3, 9_ |  |  | 60780.72 | 0.0051 |
| 12_3, 9_ |  | 11_3, 8_ |  |  | 60900.52 | 0.0477 |
| 12_4, 9_ |  | 11_4, 8_ |  |  | 60753.92 | 0.0568 |
| 12_4, 8_ |  | 11_4, 7_ |  |  | 60757.91 | 0.0189 |
| 12_5, 8_ |  | 11_5, 7_ |  |  | 60721.53 | 0.0726 |
| 12_5, 7_ |  | 11_5, 6_ |  |  | 60721.53 | 0.0060 |
| 12_6, 7_ |  | 11_6, 6_ |  |  | 60704.91 | 0.0760 |
| 12_6, 6_ |  | 11_6, 5_ |  |  | 60704.91 | 0.0754 |
| 12_7, 6_ |  | 11_7, 5_ |  |  | 60696.40 | 0.0812 |
| 12_7, 5_ |  | 11_7, 4_ |  |  | 60696.40 | 0.0812 |
| 12_8, 5_ |  | 11_8, 4_ |  |  | 60692.23 | 0.0205 |
| 12_8, 4_ |  | 11_8, 3_ |  |  | 60692.23 | 0.0205 |
| 13_0, 13_ |  | 12_1, 12_ |  |  | 61020.53 | 0.0332 |
| 13_1, 13_ |  | 12_1, 12_ |  |  | 63694.82 | 0.0257 |
| 13_1, 13_ |  | 12_0, 12_ |  |  | 66905.58 | 0.0294 |
| 13_2, 12_ |  | 12_2, 11_ |  |  | 65414.27 | -0.0314 |
| 13_2, 11_ |  | 12_2, 10_ |  |  | 66874.54 | -0.0510 |
| 13_3, 11_ |  | 12_3, 10_ |  |  | 65855.55 | -0.0078 |
| 13_3, 10_ |  | 12_3, 9_ |  |  | 66032.94 | -0.0524 |
| 13_4, 10_ |  | 12_4, 9_ |  |  | 65831.98 | -0.0207 |
| 13_4, 9_ |  | 12_4, 8_ |  |  | 65839.10 | -0.0330 |
| 13_5, 9_ |  | 12_5, 8_ |  |  | 65791.38 | 0.0596 |
| 13_5, 8_ |  | 12_5, 7_ |  |  | 65791.38 | -0.0817 |
| 13_6, 8_ |  | 12_6, 7_ |  |  | 65769.76 | -0.0201 |
| 13_6, 7_ |  | 12_6, 6_ |  |  | 65769.76 | -0.0217 |
| 13_7, 7_ |  | 12_7, 6_ |  |  | 65758.38 | -0.0790 |
| 13_7, 6_ |  | 12_7, 5_ |  |  | 65758.38 | -0.0790 |
| 13_8, 6_ |  | 12_8, 5_ |  |  | 65752.68 | 0.0144 |
| 13_8, 5_ |  | 12_8, 4_ |  |  | 65752.68 | 0.0144 |
| 13_9, 4_ |  | 12_9, 3_ |  |  | 65750.21 | -0.0053 |
| 13_9, 5_ |  | 12_9, 4_ |  |  | 65750.21 | -0.0053 |
| 14_0, 14_ |  | 13_0, 13_ |  |  | 69015.82 | 0.0762 |
| 14_1, 13_ |  | 13_1, 12_ |  |  | 71826.91 | -0.0355 |
| 14_2, 13_ |  | 13_2, 12_ |  |  | 70393.90 | -0.0403 |
| 14_3, 12_ |  | 13_3, 11_ |  |  | 70929.19 | -0.0264 |
| 14_3, 11_ |  | 13_3, 10_ |  |  | 71183.28 | 0.0141 |
| 14_4, 11_ |  | 13_4, 10_ |  |  | 70913.29 | -0.0264 |
| 14_4, 10_ |  | 13_4, 9_ |  |  | 70925.35 | -0.0318 |
| 14_8, 7_ |  | 13_8, 6_ |  |  | 70813.73 | -0.0548 |
| 14_8, 6_ |  | 13_8, 5_ |  |  | 70813.73 | -0.0548 |
| 17_4, 13_ |  | 17_3, 14_ |  |  | 64967.22 | 0.0436 |
| 16_4, 13_ |  | 16_3, 14_ |  |  | 66989.14 | -0.0142 |
| 16_4, 12_ |  | 16_3, 13_ |  |  | 65503.73 | 0.0341 |
| 15_4, 12_ |  | 15_3, 13_ |  |  | 66972.34 | -0.0047 |
| 15_4, 11_ |  | 15_3, 12_ |  |  | 65932.62 | -0.0087 |
| 14_4, 11_ |  | 14_3, 12_ |  |  | 66975.13 | -0.0119 |
| 14_4, 11_ |  | 14_3, 11_ |  |  | 66241.34 | -0.0082 |
| 14_4, 10_ |  | 14_3, 11_ |  |  | 66268.61 | -0.0112 |
| 13_4, 10_ |  | 13_3, 11_ |  |  | 66991.04 | -0.0019 |
| 12_4, 9_ |  | 12_3, 10_ |  |  | 67014.60 | 0.0010 |
| 12_4, 8_ |  | 12_3, 9_ |  |  | 66720.37 | 0.0052 |
| 11_4, 7_ |  | 11_3, 9_ |  |  | 67045.50 | 0.0019 |
| 11_4, 8_ |  | 11_3, 8_ |  |  | 66858.83 | -0.0686 |
| 11_4, 7_ |  | 11_3, 8_ |  |  | 66862.96 | 0.0140 |

Table S2. Experimental transition frequencies of MAE conformer *gG’T* isotopologue ^13^C_1

| *J”_K”aK”c_* | *F* | *J'_K’aK’c_* | *F'* | *ν*_OBS_/MHz | ∆*ν*_OBS-CALC_/MHz |
| --- | --- | --- | --- | --- | --- |
|  | 2F |  | 2F |  |  |
| 2_0,2_ | 6 | 1_0,1_ | 4 | 9849.2832 | 0.0057 |
| 2_1,1_ | 6 | 1_1,0_ | 4 | 10094.5166 | -0.0054 |
|  | *4* |  | 2 | 10095.0871 | 0.0185 |
|  | 4 |  | 4 | 10095.6175 | -0.0036 |
| 2_1,2_ | 6 | 1_1,1_ | 4 | 9613.0181 | -0.0053 |
|  | *4* |  | 2 | 9613.4547 | -0.0025 |
|  | 4 |  | 4 | 9612.4403 | 0.0089 |
| 3_0,3_ | 6 | 2_0,2_ | 4 | 14762.6030 | 0.0008 |
|  | 8 |  | 6 | 14762.6265 | 0.0093 |
|  | 4 |  | 2 | 14762.7146 | -0.0078 |
| 3_1,2_ | 6 | 2_1,1_ | 4 | 15139.1708 | -0.0040 |
|  | 8 |  | 6 | 15139.0061 | -0.0006 |
|  | 4 |  | 2 | 15139.1708 | -0.0011 |

Table S3. Experimental transition frequencies of MAE conformer *gG’T* isotopologue ^13^C_3

| *J”_K”aK”c_* | *F* | *J'_K’aK’c_* | *F'* | *ν*_OBS_/MHz | ∆*ν*_OBS-CALC_/MHz |
| --- | --- | --- | --- | --- | --- |
| 2_0,2_ | 6 | 1_0,1_ | 4 | 10088.5338 | -0.0006 |
|  | 4 |  | 4 | 10089.0173 | -0.0008 |
| 2_1,1_ | 6 | 1_1,0_ | 4 | 10352.8419 | 0.0048 |
| 2_1,2_ | 6 | 1_1,1_ | 4 | 9834.8571 | -0.0004 |
|  | 4 |  | 2 | 9835.2917 | 0.0004 |
| 3_0,3_ | 6 | 2_0,2_ | 4 | 15119.3979 | -0.0001 |
|  | 8 |  | 6 | 15119.4186 | 0.0021 |
|  | 4 |  | 2 | 15119.5212 | -0.0011 |
| 3_1,2_ | 6 | 2_1,1_ | 4 | 15526.1167 | -0.0005 |
|  | 8 |  | 6 | 15525.9544 | 0.0017 |
|  | 4 |  | 2 | 15526.1167 | -0.0044 |

Table S4. Experimental transition frequencies of MAE conformer *gG’T* isotopologue ^13^C_4

| *J”_K”aK”c_* | *F* | *J'_K’aK’c_* | *F'* | *ν*_OBS_/MHz | ∆*ν*_OBS-CALC_/MHz |
| --- | --- | --- | --- | --- | --- |
| 2_0,2_ | 6 | 1_0,1_ | 4 | 10002.4142 | 0.0006 |
| 2_1,1_ | 6 | 1_1,0_ | 4 | 10258.4026 | 0.0009 |
| 2_1,2_ | 6 | 1_1,1_ | 4 | 9756.2833 | -0.0003 |
| 3_0,3_ | 6 | 2_0,2_ | 4 | 14991.1746 | -0.0015 |
|  | 8 |  | 6 | 14991.1943 | 0.0013 |
|  | 4 |  | 2 | 14991.2987 | 0.0002 |
| 3_1,2_ | 6 | 2_1,1_ | 4 | 15384.5371 | -0.0065 |
|  | 4 |  | 2 | 15384.7175 | 0.0057 |

Table S5. Experimental transition frequencies of MAE conformer *gG’T* isotopologue ^15^N

| *J”_K”aK”c_* | *F* | *J'_K’aK’c_* | *F'* | *ν*_OBS_/MHz | ∆*ν*_OBS-CALC_/MHz |
| --- | --- | --- | --- | --- | --- |
| 2_0,2_ |  | 1_0,1_ |  | 10058.4979 | 0.0002 |
| 2_1,1_ |  | 1_1,0_ |  | 10313.2676 | -0.0007 |
| 3_0,3_ |  | 2_0,2_ |  | 15075.4428 | -0.0001 |
| 3_1,2_ |  | 2_1,1_ |  | 15466.7277 | 0.0005 |

Table S6. Experimental transition frequencies of MAE conformer *gG’T* isotopologue CH(OD)CHN(D)CH_3_

| *J'* | | *K’a* | | *K’c* | | *F'* | | *J''* | | *K”a* | | *K”c* | | *F”* | | ν_OBS/MHz_ | | ∆ν_OBS-CALC_/MHz | |
| --- | --- | --- | --- | --- | --- | --- | --- | --- | --- | --- | --- | --- | --- | --- | --- | --- | --- | --- | --- |
| 4 | | 3 | | 2 | | - | | 3 | | 2 | | 1 | | - | | 63193.14 | | -0.06 | |
| 4 | | 3 | | 1 | | - | | 3 | | 2 | | 1 | | - | | 63193.82 | | 0.14 | |
| 4 | | 3 | | 1 | | - | | 3 | | 2 | | 2 | | - | | 63219.51 | | 0.18 | |
| 4 | | 3 | | 2 | | - | | 3 | | 2 | | 2 | | - | | 63218.85 | | -0.01 | |
| 5 | | 3 | | 3 | | - | | 4 | | 2 | | 2 | | - | | 68128.88 | | -0.09 | |
| 5 | | 3 | | 2 | | - | | 4 | | 2 | | 2 | | - | | 68130.84 | | -0.02 | |
| 5 | | 3 | | 2 | | - | | 4 | | 2 | | 3 | | - | | 68207.76 | | 0.03 | |
| 5 | | 3 | | 3 | | - | | 4 | | 2 | | 3 | | - | | 68205.76 | | -0.07 | |
| 6 | | 3 | | 4 | | - | | 5 | | 2 | | 3 | | - | | 73030.63 | | -0.08 | |
| 6 | | 3 | | 3 | | - | | 5 | | 2 | | 3 | | - | | 73036.38 | | 0.00 | |
| 6 | | 3 | | 3 | | - | | 5 | | 2 | | 4 | | - | | 73215.25 | | 0.01 | |
| 6 | | 3 | | 4 | | - | | 5 | | 2 | | 4 | | - | | 73209.60 | | 0.03 | |
| 7 | | 2 | | 5 | | 6 | | 6 | | 1 | | 6 | | 5 | | 63957.26 | | -0.01 | |
|  | |  | |  | | 8 | |  | |  | |  | | 7 | |  |  |  |  |
|  | |  | |  | | 7 | |  | |  | |  | | 6 | | 63958.53 | | 0.01 | |
| 7 | | 2 | | 6 | | 6 | | 6 | | 1 | | 6 | | 5 | | 63321.17 | | -0.03 | |
|  | |  | |  | | 8 | |  | |  | |  | | 7 | |  |  |  |  |
|  | |  | |  | | 7 | |  | |  | |  | | 6 | | 63322.06 | | -0.03 | |
| 12 | | 1 | | 11 | | - | | 11 | | 1 | | 10 | | - | | 60617.05 | | 0.01 | |
| 12 | | 2 | | 10 | | - | | 11 | | 2 | | 9 | | - | | 60572.17 | | 0.09 | |
| 13 | | 0 | | 13 | | - | | 12 | | 1 | | 12 | | - | | 60378.21 | | 0.02 | |
| 13 | | 2 | | 12 | | - | | 12 | | 2 | | 11 | | - | | 64255.32 | | -0.06 | |
| 13 | | 3 | | 11 | | - | | 12 | | 3 | | 10 | | - | | 64702.66 | | -0.04 | |
| 13 | | 3 | | 10 | | - | | 12 | | 3 | | 9 | | - | | 64895.71 | | -0.09 | |
| 13 | | 4 | | 10 | | - | | 12 | | 4 | | 9 | | - | | 64682.73 | | 0.04 | |
| 13 | | 4 | | 9 | | - | | 12 | | 4 | | 8 | | - | | 64691.03 | | 0.03 | |
| 14 | | 0 | | 14 | | - | | 13 | | 1 | | 13 | | - | | 65555.72 | | 0.06 | |
| 14 | | 2 | | 12 | | - | | 13 | | 2 | | 11 | | - | | 70864.72 | | -0.01 | |
| 15 | | 0 | | 15 | | - | | 14 | | 1 | | 14 | | - | | 70664.65 | | -0.02 | |
| 4 | | 4 | | 1 | | - | | 4 | | 3 | | 2 | | - | | 60663.91 | | 0.00 | |
| 4 | | 4 | | 0 | | - | | 4 | | 3 | | 1 | | - | |  |  |  |  |
| 4 | | 4 | | 0 | | - | | 4 | | 3 | | 2 | | - | |  |  |  |  |
| 4 | | 4 | | 1 | | - | | 4 | | 3 | | 1 | | - | |  |  |  |  |
| 5 | | 4 | | 2 | | - | | 5 | | 3 | | 3 | | - | | 60658.91 | | -0.12 | |
| 5 | | 4 | | 1 | | - | | 5 | | 3 | | 3 | | - | |  |  |  |  |
| 5 | | 4 | | 2 | | - | | 5 | | 3 | | 2 | | - | | 60657.17 | | 0.03 | |
| 5 | | 4 | | 1 | | - | | 5 | | 3 | | 2 | | - | |  |  |  |  |
| 6 | | 4 | | 3 | | - | | 6 | | 3 | | 4 | | - | | 60650.54 | | -0.08 | |
| 6 | | 4 | | 2 | | - | | 6 | | 3 | | 4 | | - | |  |  |  |  |
| 6 | | 4 | | 3 | | - | | 6 | | 3 | | 3 | | - | | 60644.97 | | 0.02 | |
| 6 | | 4 | | 2 | | - | | 6 | | 3 | | 3 | | - | |  |  |  |  |
| 7 | | 4 | | 4 | | - | | 7 | | 3 | | 4 | | - | | 60624.16 | | 0.04 | |
| 7 | | 4 | | 3 | | - | | 7 | | 3 | | 4 | | - | |  |  |  |  |
| 7 | | 4 | | 4 | | - | | 7 | | 3 | | 5 | | - | | 60638.24 | | -0.03 | |
| 7 | | 4 | | 3 | | - | | 7 | | 3 | | 5 | | - | |  |  |  |  |
| 8 | | 4 | | 5 | | - | | 8 | | 3 | | 5 | | - | | 60590.68 | | 0.07 | |
| 8 | | 4 | | 4 | | - | | 8 | | 3 | | 5 | | - | |  |  |  |  |
| 8 | | 4 | | 5 | | - | | 8 | | 3 | | 6 | | - | | 60621.70 | | 0.08 | |
| 8 | | 4 | | 4 | | - | | 8 | | 3 | | 6 | | - | |  |  |  |  |
| 9 | | 4 | | 6 | | - | | 9 | | 3 | | 7 | | - | | 60600.58 | | -0.10 | |
| 9 | | 4 | | 5 | | - | | 9 | | 3 | | 7 | | - | | 60601.63 | | 0.01 | |
| 10 | | 4 | | 7 | | - | | 10 | | 3 | | 8 | | - | | 60576.53 | | -0.02 | |
| 10 | | 4 | | 7 | | - | | 10 | | 3 | | 7 | | - | | 60462.00 | | 0.06 | |
| 10 | | 4 | | 6 | | - | | 10 | | 3 | | 8 | | - | | 60578.78 | | 0.02 | |
| 10 | | 4 | | 6 | | - | | 10 | | 3 | | 7 | | - | | 60464.20 | | 0.05 | |
| 11 | | 4 | | 7 | | - | | 11 | | 3 | | 9 | | - | | 60555.52 | | 0.00 | |
| 11 | | 4 | | 7 | | - | | 11 | | 3 | | 8 | | - | | 60356.01 | | -0.06 | |

Table S7. Experimental transition frequencies of MAE conformer *gG’T* isotopologue CH(OH)CHN(D)CH_3_

| *J'* | *K’a* | *K’c* | *F'* | *J”* | *K”a* | *K”c* | *F”* | ν_OBS_/MHz | ∆ν_OBS-CALC_/MHz |
| --- | --- | --- | --- | --- | --- | --- | --- | --- | --- |
| 3 | 3 | 1 | - | 2 | 2 | 0 | - | 60562.13 | 0.07 |
| 3 | 3 | 0 | - | 2 | 2 | 0 | - |  |  |
| 3 | 3 | 0 | - | 2 | 2 | 1 | - | 60566.18 | 0.07 |
| 3 | 3 | 1 | - | 2 | 2 | 1 | - |  |  |
| 4 | 3 | 2 | - | 3 | 2 | 1 | - | 65561.59 | 0.09 |
| 4 | 3 | 1 | - | 3 | 2 | 1 | - | 65562.11 | 0.09 |
| 4 | 3 | 1 | - | 3 | 2 | 2 | - | 65583.04 | 0.09 |
| 4 | 3 | 2 | - | 3 | 2 | 2 | - | 65582.53 | 0.08 |
| 5 | 3 | 3 | - | 4 | 2 | 2 | - | 70544.28 | 0.08 |
| 5 | 3 | 2 | - | 4 | 2 | 2 | - | 70545.79 | 0.07 |
| 5 | 3 | 2 | - | 4 | 2 | 3 | - | 70608.52 | 0.09 |
| 5 | 3 | 3 | - | 4 | 2 | 3 | - | 70606.98 | 0.09 |
| 7 | 2 | 6 | 6 | 6 | 1 | 5 | 5 | 60009.68 | 0.00 |
|  |  |  | 8 |  |  |  | 7 |  |  |
|  |  |  | 7 |  |  |  | 6 | 60008.54 | 0.00 |

Table S8. Experimental transition frequencies of MAE conformer *gG’T* isotopologue CH(OD)CHN(H)CH_3_

| *J'* | *K’a* | *K’c* | *F'* | *J”* | *K”a* | *K”c* | *F”* | ν_OBS_/MHz | ∆_νOBS-CALC_/MHz |
| --- | --- | --- | --- | --- | --- | --- | --- | --- | --- |
| 3 | 3 | 1 | - | 2 | 2 | 0 | - | 60610.91 | -0.02 |
| 3 | 3 | 0 | - | 2 | 2 | 0 | - |  |  |
| 3 | 3 | 0 | - | 2 | 2 | 1 | - | 60616.94 | -0.04 |
| 3 | 3 | 1 | - | 2 | 2 | 1 | - |  |  |
| 4 | 3 | 2 | - | 3 | 2 | 1 | - | 65611.93 | -0.12 |
| 4 | 3 | 1 | - | 3 | 2 | 1 | - | 65612.72 | 0.08 |
| 4 | 3 | 1 | - | 3 | 2 | 2 | - | 65642.95 | 0.10 |
| 4 | 3 | 2 | - | 3 | 2 | 2 | - | 65642.14 | -0.12 |
| 5 | 3 | 3 | - | 4 | 2 | 2 | - | 70589.79 | 0.00 |
| 5 | 3 | 2 | - | 4 | 2 | 2 | - | 70592.27 | 0.12 |
| 5 | 3 | 2 | - | 4 | 2 | 3 | - | 70682.71 | 0.07 |
| 5 | 3 | 3 | - | 4 | 2 | 3 | - | 70680.23 | -0.06 |
| 8 | 2 | 7 | 7 | 7 | 1 | 6 | 6 | 63669.17 | 0.04 |
|  |  |  | 9 |  |  |  | 8 |  |  |
|  |  |  | 8 |  |  |  | 7 | 63668.11 | -0.08 |
| 8 | 2 | 6 | 7 | 7 | 1 | 7 | 6 | 72473.11 | 0.05 |
|  |  |  | 9 |  |  |  | 8 |  |  |
|  |  |  | 8 |  |  |  | 7 | 72474.29 | -0.04 |
| 12 | 1 | 11 | - | 11 | 1 | 10 | - | 61269.91 | -0.09 |
| 12 | 2 | 11 | - | 11 | 2 | 10 | - | 59883.71 | 0.09 |
| 13 | 1 | 13 | - | 12 | 0 | 12 | - | 65624.99 | -0.03 |
| 13 | 2 | 12 | - | 12 | 2 | 11 | - | 64819.82 | 0.01 |
| 14 | 0 | 14 | - | 13 | 0 | 13 | - | 68183.34 | 0.07 |
| 14 | 3 | 11 | - | 13 | 3 | 10 | - | 70714.09 | -0.05 |

Table S9. Experimental transition frequencies of MAE conformer *g’GG*

| *J”_K”aK”c_* | *F* | *J'_K’aK’c_* | *F'* |  | *ν*_OBS_/MHz | ∆*ν*_OBS-CALC_/MHz |
| --- | --- | --- | --- | --- | --- | --- |
| 2_0,2_ | 2 | 1_0,1_ | 2 |  | 11884.3088 | -0.0032 |
|  | 4 |  | 2 |  | 11884.5285 | -0.0063 |
|  | 6 |  | 4 |  | 11884.5467 | 0.0022 |
| 2_1,1_ | 2 | 1_0,0_ | 2 |  | 12096.5745 | -0.0006 |
|  | 2 |  | 4 |  | 12097.0689 | -0.0008 |
|  | 6 |  | 4 |  | 12097.4551 | 0.0000 |
|  | 4 |  | 2 |  | 12097.6524 | -0.0018 |
|  | 2 |  | 0 |  | 12097.8090 | -0.0025 |
|  | 4 |  | 4 |  | 12098.1478 | -0.0009 |
| 2_1,2_ | 2 | 1_1,1_ | 0 |  | 11681.5975 | 0.0011 |
|  | 4 |  | 4 |  | 11681.7363 | -0.0069 |
|  | 6 |  | 4 |  | 11682.2708 | -0.0023 |
|  | 4 |  | 2 |  | 11682.3858 | -0.0048 |
|  | 2 |  | 4 |  | 11682.5681 | 0.0006 |
|  | 2 |  | 2 |  | 11683.2154 | 0.0005 |
| 3_0,3_ | 4 | 2_0,2_ | 6 |  | 17813 4898 | 0.0061 |
|  | 8 |  | 6 |  | 17813 5255 | 0.001 |
|  | 4 |  | 2 |  | 17813.5639 | 0.0007 |
| 3_1,2_ | 8 | 2_1,1_ | 6 |  | 18142.6289 | -0.0009 |
|  | 6 |  | 4 |  | 18142.6893 | -0.0018 |
| 3_1,3_ | 6 | 2_1,2_ | 6 |  | 17519.4565 | -0.0031 |
|  | 6 |  | 4 |  | 17519.9909 | 0.0014 |
|  | 4 |  | 4 |  | 17520.6755 | -0.0074 |
| 1_1,1_ | 2 | 0_0,0_ | 2 | E | 12023.3744 | -0.0022 |
|  |  |  |  | A | 12023.3923 | -0.0007 |
|  | 4 |  | 2 | E | 12024.0223 | -0.0017 |
|  |  |  |  | A | 12024.0399 | -0.0005 |
|  | 0 |  | 2 | E | 12024.9953 | 0.0001 |
|  |  |  |  | A | 12025.0103 | -0.0013 |
| 1_1,0_ | 0 | 0_0,0_ | 2 | E | 12230.7505 | 0.0028 |
|  |  |  |  | A | 12230.7630 | 0.0008 |
|  | 4 |  | 2 | E | 12231.4904 | 0.0008 |
|  |  |  |  | A | 12231.5093 | 0.0052 |
|  | 2 |  | 2 | E | 12231.9837 | -0.0005 |
|  |  |  |  | A | 12232.0026 | 0.0040 |
| 4_1,3_ | 10 | 4_0,4_ | 10 |  | 7285.0649 | 0.0039 |
|  | 8 |  | 8 |  | 7285.2385 | -0.0032 |
|  | 8 |  | 10 |  | 7285.9485 | 0.0039 |
| 5_1,4_ | 10 | 5_0,5_ | 10 |  | 7884.6935 | 0.0051 |
|  | 10 |  | 12 |  | 7884.7029 | -0.0087 |
|  | 8 |  | 8 |  | 7883.7714 | 0.0000 |
|  | 12 |  | 12 |  | 7883.9329 | 0.0064 |
| 2_1,2_ | 4 | 1_0,1_ | 2 | E | 17760.6922 | -0.0024 |
|  |  |  |  | A | 17760.7125 | -0.0018 |
|  | 4 |  | 4 | E | 17760.8447 | -0.0027 |
|  |  |  |  | A | 17760.8656 | -0.0015 |
|  | 6 |  | 4 | E | 17761.3770 | -0.0003 |
|  |  |  |  | A | 17761.3974 | 0.0004 |
|  | 2 |  | 2 | E | 17761.5120 | -0.0069 |
|  |  |  |  | A | 17761.5326 | -0.006 |
|  | 2 |  | 0 | E | 17761.9041 | 0.0031 |
|  |  |  |  | A | 17761.9259 | 0.0052 |
| 3_2,2_ | 4 | 2_2,1_ | 2 | A | 17834.9739 | 0.0003 |
|  | 4 |  | 2 | E | 17834.9958 | 0.0011 |
|  | 8 |  | 6 | A | 17835.0685 | 0.0040 |
|  | 8 |  | 6 | E | 17835.0878 | 0.0022 |
|  | 6 |  | 4 | A | 17835.2289 | 0.0006 |
|  | 6 |  | 4 | E | 17835.2454 | -0.0039 |
| 3_2,1_ | 4 | 2_2,0_ | 2 | E | 17855.8206 | 0.0088 |
|  |  |  |  | A | 17855.8565 | 0.0117 |
|  | 8 |  | 6 | E | 17855.9043 | -0.0058 |
|  |  |  |  | A | 17855.9446 | 0.0015 |
|  | 4 |  | 4 | E | 17856.1063 | -0.0004 |
|  |  |  |  | A | 17856.1390 | -0.0006 |
| 3_0,3_ | 4 | 2_1,2_ | 6 | A | 11936.3385 | 0.0018 |
|  |  |  |  | E | 11936.3431 | -0.0026 |
|  | 8 |  | 6 | A | 11936.6721 | 0.0002 |
|  |  |  |  | E | 11936.6801 | -0.0008 |
|  | 6 |  | 4 | A | 11937.3232 | 0.0049 |
|  |  |  |  | E | 11937.3286 | 0.0013 |
| 10_2,8_ |  | 9_2,7_ |  |  | 60053.02 | 0.05 |
| 10_1,9_ |  | 9_1,8_ |  |  | 60226.55 | 0.01 |
| 10_1,10_ | 20 | 9_0,9_ | 18 |  | 61000.62 | -0.03 |
|  | 22 |  | 20 |  | 61001.01 | -0.00 |
|  | 18 |  | 16 |  | 61001.01 | -0.04 |
| 11_0,11_ |  | 10 _1,10_ |  |  | 62100.97 | -0.00 |
| 11_1,11_ |  | 10 _1,10_ |  |  | 64004.76 | -0.02 |
| 11_0,11_ |  | 10 _0,10_ |  |  | 64420.61 | -0.02 |
| 11_2,10_ |  | 10 _2,9_ |  |  | 65197.77 | 0.07 |
| 11_6,6_ |  | 10 _6,5_ |  |  | 65436.74 | 0.03 |
| 11_6,5_ |  | 10 _6,4_ |  |  | 65436.74 | 0.03 |
| 11_8,3_ |  | 10 _8,2_ |  |  | 65439.98 | 0.11 |
| 11_8,4_ |  | 10 _8,3_ |  |  | 65439.98 | 0.11 |
| 11_5,7_ |  | 10 _5,6_ |  |  | 65444.85 | 0.04 |
| 11_5,6_ |  | 10 _5,5_ |  |  | 65444.85 | -0.02 |
| 11_4,8_ |  | 10 _4,7_ |  |  | 65466.37 | 0.04 |
| 11_4,7_ |  | 10 _4,6_ |  |  | 65469.89 | -0.05 |
| 11_3,9_ |  | 10 _3,8_ |  |  | 65482.21 | 0.06 |
| 11_3,8_ |  | 10 _3,7_ |  |  | 65583.47 | -0.05 |
| 11_2,9_ |  | 10 _2,8_ |  |  | 66144.82 | -0.08 |
| 11_1,10_ |  | 1 _1,9_ |  |  | 66177.79 | 0.03 |
| 11_1,11_ | 22 | 10 _0,10_ | 20 |  | 66324.26 | 0.03 |
|  | 24 |  | 22 |  | 66324.51 | -0.02 |
|  | 20 |  | 18 |  | 66324.51 | -0.05 |
| 12_0,12_ |  | 11 _1,11_ |  |  | 68241.77 | -0.02 |
| 12_1,12_ |  | 11 _1,11_ |  |  | 69781.26 | -0.04 |
| 12_0,12_ |  | 11 _0,11_ |  |  | 70145.69 | 0.09 |
| 12_2,11_ |  | 11 _2,10_ |  |  | 71081.66 | 0.03 |
| 12_7,5_ |  | 11 _7,4_ |  |  | 71384.94 | 0.04 |
| 12_7,6_ |  | 11 _7,5_ |  |  | 71384.94 | 0.04 |
| 12_6,7_ |  | 11 _6,6_ |  |  | 71387.51 | 0.05 |
| 12_6,6_ |  | 11 _6,5_ |  |  | 71387.51 | 0.05 |
| 12_8,4_ |  | 11 _8,3_ |  |  | 71387.90 | 0.01 |
| 12_8,5_ |  | 11 _8,4_ |  |  | 71387.90 | 0.01 |
| 12_9,3_ |  | 11 _9,2_ |  |  | 71394.67 | 0.03 |
| 12_9,4_ |  | 11 _9,3_ |  |  | 71394.67 | 0.03 |
| 12_5,8_ |  | 11 _5,7_ |  |  | 71399.45 | 0.06 |
| 12_5,7_ |  | 11 _5,6_ |  |  | 71399.45 | -0.08 |
| 12_4,9_ |  | 11 _4,8_ |  |  | 71427.53 | -0.08 |
| 12_4,8_ |  | 11 _4,7_ |  |  | 71434.23 | -0.10 |
| 12_3,10_ |  | 11 _3,9_ |  |  | 71438.87 | -0.08 |
| 12_3,9_ |  | 11 _3,8_ |  |  | 71593.89 | -0.07 |
| 7_2,5_ | 12 | 6 _1,6_ | 10 |  | 62977.30 | 0.04 |
|  | 16 |  | 14 |  | 62977.30 | -0.11 |
|  | 14 |  | 12 |  | 62978.30 | -0.05 |
| 8_2,7_ | 16 | 7_1,6_ | 14 |  | 63212.25 | 0.05 |
|  | 18 |  | 16 |  | 63212.98 | 0.11 |
|  | 14 |  | 12 |  | 63212.98 | 0.01 |
| 5_3,3_ |  | 4_2,2_ |  |  | 60593.22 | -0.07 |
| 5_3,2_ |  | 4_2,2_ |  |  | 60595.81 | 0.22 |
| 5_3,3_ |  | 4_2,3_ |  |  | 60671.26 | -0.20 |
| 5_3,2_ |  | 4_2,3_ |  |  | 60673.81 | 0.05 |
| 6_3,4_ |  | 5_2,3_ |  |  | 66473.20 | -0.04 |
| 6_3,4_ |  | 5_2,4_ |  |  | 66654.77 | -0.09 |
| 6_3,3_ |  | 5_2,4_ |  |  | 66661.74 | 0.00 |
| 9_2,8_ | 18 | 8_1,7_ | 16 |  | 68347.71 | 0.09 |
|  | 20 |  | 18 |  | 68348.42 | 0.14 |
|  | 16 |  | 14 |  | 68348.42 | 0.06 |
| 8_2,6_ | 14 | 7_1,7_ | 12 |  | 70058.17 | -0.01 |
|  | 18 |  | 16 |  | 70058.17 | -0.15 |
|  | 16 |  | 14 |  | 70059.27 | -0.08 |
| 12_1,12_ | 24 | 11_0,11_ | 22 |  | 71684.92 | -0.02 |
|  | 26 |  | 24 |  | 71685.15 | -0.04 |
|  | 22 |  | 20 |  | 71685.16 | -0.05 |
| 4_4,0_ |  | 3_3,0_ |  |  | 67050.82 | -0.01 |
| 14_1,13_ |  | 13_2,11_ |  |  | 67911.36 | -0.07 |
| 7_3,5_ |  | 6_2,4_ |  |  | 72301.04 | 0.08 |
| 7_3,4_ |  | 6_2,4_ |  |  | 72678.89 | 0.07 |
| 5_4_ |  | 4_3_ |  |  | 72998.43 | 0.02 |
| 14 _6_ |  | 14 _5_ |  |  | 67889.02 | -0.06 |
| 13 _6_ |  | 13 _5_ |  |  | 67911.36 | 0.00 |
| 12 _6_ |  | 12 _5_ |  |  | 67928.04 | -0.01 |
| 11 _6_ |  | 11 _5_ |  |  | 67940.01 | -0.05 |
| 10 _6_ |  | 10 _5_ |  |  | 67948.12 | -0.07 |
| 9 _6_ |  | 9 _5_ |  |  | 67953.14 | -0.06 |
| 8 _6_ |  | 8 _5_ |  |  | 67956.01 | 0.25 |

Table S10. Experimental transition frequencies of MAE conformer *g’GG* isotopologue ^13^C_1

| *J”_K”aK”c_* | *F* | *J'_K’aK’c_* | *F'* | *ν*_OBS_/MHz | ∆*ν*_OBS-CALC_/MHz |
| --- | --- | --- | --- | --- | --- |
| 2_0,2_ | 6 | 1_0,1_ | 4 | 11626.0147 | -0.0029 |
| 2_1,1_ | 6 | 1_1,0_ | 4 | 11830.4437 | -0.0082 |
|  | 4 |  | 2 | 11830.6590 | 0.0080 |
| 2_1,2_ | 6 | 1_1,1_ | 4 | 11431.4149 | -0.0073 |
| 3_0,3_ | 8 | 2_0,2_ | 6 | 17426.7309 | -0.0004 |
| 3_1,2_ | 6 | 2_1,1_ | 4 | 17742.4413 | -0.0003 |
|  | 8 |  | 6 | 17742.3816 | 0.0016 |
| 3_1,3_ | 6 | 2_1,2_ | 4 | 17143.9678 | 0.0088 |
|  | 8 |  | 6 | 17143.9398 | -0.0027 |
|  |  |  |  |  |  |

Table S11. Experimental transition frequencies of MAE conformer *g’GG* isotopologue ^13^C_3

| *J”_K”aK”c_* | *F* | *J'_K’aK’c_* | *F'* | *ν*_OBS_/MHz | ∆*ν*_OBS-CALC_/MHz |
| --- | --- | --- | --- | --- | --- |
| 2_0,2_ | 6 | 1_0,1_ | 4 | 11851.7557 | -0.0016 |
| 2_1,1_ | 6 | 1_1,0_ | 4 | 12080.8036 | -0.0035 |
| 2_1,2_ | 6 | 1_1,1_ | 4 | 11635.2290 | -0.0030 |
|  | 4 |  | 4 | 11634.7001 | -0.0020 |
| 3_0,3_ | 6 | 2_0,2_ | 4 | 17761.9758 | 0.0006 |
|  | 8 |  | 6 | 17762.0047 | 0.0004 |
| 3_1,2_ | 6 | 2_1,1_ | 4 | 18117.1070 | -0.0013 |
|  | 8 |  | 6 | 18117.0471 | -0.0006 |
| 3_1,3_ | 8 | 2_1,2_ | 6 | 17448.8402 | 0.0038 |

Table S12. Experimental transition frequencies of MAE conformer *g’GG* isotopologue ^13^C_4

| *J”_K”aK”c_* | *F* | *J'_K’aK’c_* | *F'* | *ν*_OBS_/MHz | ∆*ν*_OBS-CALC_/MHz |
| --- | --- | --- | --- | --- | --- |
| 2_0,2_ | 6 | 1_0,1_ | 4 | 11781.7992 | -0.0008 |
| 2_1,1_ | 6 | 1_1,0_ | 4 | 11986.7688 | -0.0057 |
|  | 2 |  | 0 | 11987.1235 | -0.0074 |
| 2_1,2_ | 6 | 1_1,1_ | 4 | 11586.7758 | -0.0029 |
|  | 4 |  | 2 | 11586.9009 | 0.0046 |
| 3_0,3_ | 6 | 2_0,2_ | 4 | 17660.2308 | -0.0062 |
|  | 8 |  | 6 | 17660.2675 | 0.0049 |
| 3_1,2_ | 6 | 2_1,1_ | 4 | 17976.8929 | 0.0041 |
|  | 8 |  | 6 | 17976.8307 | 0.0034 |
|  | 4 |  | 2 | 17976.9505 | 0.0022 |
| 3_1,3_ | 8 | 2_1,2_ | 6 | 17376.9421 | -0.0002 |

Table S13. Experimental transition frequencies (MHz) of MAE conformer *g’GG* isotopologue ^15^N

| *J”_K”aK”c_* | *F* | *J'_K’aK’c_* | *F'* | *ν*_OBS_/MHz | ∆*ν*_OBS-CALC_/MHz |
| --- | --- | --- | --- | --- | --- |
| 2_0,2_ |  | 1_0,1_ |  | 11807.4088 | 0.0019 |
| 2_1,1_ |  | 1_1,0_ |  | 12011.0575 | -0.0048 |
| 2_1,2_ |  | 1_1,1_ |  | 11613.6187 | -0.0064 |
| 3_0,3_ |  | 2_0,2_ |  | 17698.8195 | -0.0017 |
| 3_1,2_ |  | 2_1,1_ |  | 18013.2305 | 0.0034 |
| 3_1,3_ |  | 2_1,2_ |  | 17417.2600 | 0.0045 |

Table S14. Spectroscopic constants of MAE conformer *gG’T* isotopologues

|  | ^13^C_1 | ^13^C_3 | ^13^C_4 | ^15^N | OD-ND | OH-ND | OD-NH |
| --- | --- | --- | --- | --- | --- | --- | --- |
| *A*/MHz | 12091.1(9)^a^ | 11936.0(2) | 12031.5(4) | 12070.6(9) | 11152.01(6)^)^ | 11612.8(1) | 11622.16(3) |
| *B*/MHz | 2583.9073(3) | 2653.0478(1) | 2627.4558(1) | 2640.8151(3) | 2604.85(1) | 2617.40(5) | 2642.27(1) |
| *C*/MHz | 2343.0286(5) | 2393.9254(2) | 2376.2652(3) | 2390.9018(7) | 2361.12(1) | 2391.80(6) | 2371.05(1) |
| *D_J_*/kHz | [1.1589]^b^ | [1.1589] | [1.1589] | [1.1589] | 1.15(4) | 9.7(5) | 1.11(4) |
| *D_JK_*/kHz | [-8.389] | [-8.389] | [-8.389] | [-8.389] | -5.7(1) | [-8.389] | -6.1(7) |
| *D_K_*/kHz | [70.88] | [70.88] | [70.88] | [70.88] | 58(2) | [70.88] | [70.88] |
| *d_1_*/kHz | [-0.2207] | [-0.2207] | [-0.2207] | [-0.2207] | -0.209(9) | [-0.2207] | [-0.2207] |
| *d_2_*/kHz | [-0.0107] | [-0.0107] | [-0.0107] | [-0.0107] | [-0.0107] | [-0.0107] | [-0.0107] |
| *χ_aa_*/MHz | [1.578] | [1.578] | [1.578] | [1.578] | [1.578] | [1.578] | [1.578] |
| *χ_bb-cc_*/MHz | [-5.262] | [-5.262] | [-5.262] | [-5.262] | -4.8(5) | -5.2(3) | -4.5(6) |
| *V_3_/*kJ*mol^-1^ | [11.91] | [11.91] | [11.91] | [11.91] | [11.91] | [11.91] | [11.91] |
| *δ/*deg | [2.7598] | [2.7598] | [2.7598] | [2.7598] | [2.7598] | [2.7598] | [2.7598] |
| *ε* /deg | [1.4853] | [1.4853] | [1.4853] | [1.4853] | [1.4853] | [1.4853] | [1.4853] |
| *σ^c^*/MHz | 0.007 | 0 .002 | 0.003 | 0.0007 | 0.1 | 0.2 | 0.1 |
| *N^d^* | 13 | 11 | 8 | 4 | 44 | 12 | 20 |

^a^ Error in parentheses in units of the last digit

^b^ In brackets values fixed to those determined for the normal species

^c^ Root-mean-square deviation of the fit.

^d^ Number of lines in the fit.

Table S15. Spectroscopic constants of MAE conformer *g’GG* isotopologues

|  | ^13^C_1 | ^13^C_3 | ^13^C_4 | ^15^N |
| --- | --- | --- | --- | --- |
| *A*/MHz | 9092.6(6)^(a)^ | 9000.4(2) | 9088.7(4) | 9095(1) |
| *B*/MHz | 3007.5495(2) | 3075.9548(1) | 3046.751(2) | 3052.4749(7) |
| *C*/MHz | 2807.9450(2) | 2853.0792(1) | 2846.6633(2) | 2853.7073(7) |
| *D_J_*/kHz | [4.341] | [4.341] | [4.341] | [4.341] |
| *D_JK_*/kHz | [-28.83] | [-28.83] | [-28.83] | [-28.83] |
| *D_K_*/kHz | [84.10] | [84.10] | [84.10] | [84.10] |
| *d_1_*/kHz | [-0.927] | [-0.927] | [-0.927] | [-0.927] |
| *d_2_*/kHz | [-0.001] | [-0.001] | [-0.001] | [-0.001] |
| *χ_aa_*/MHz | [0.510] | [0.510] | [0.510] | [0.510] |
| *χ_bb-cc_*/MHz | [-3.81] | [-3.81] | [-3.81] | [-3.81] |
| *χ_cc_*/MHz |  |  |  |  |
| *σ^c^*/MHz | 0.7 | 0.2 | 0.3 | 0.06 |
| *N^d^* | 9 | 9 | 11 | 6 |

^a^ Error in parentheses in units of the last digit

^b^ In brackets values fixed to those determined for the normal species

^c^ Root-mean-square deviation of the fit.

^d^ Number of lines in the fit.

Table S16. Principal axis coordinates of heavy atoms in MAE *gG’T*

|  | r_e_ | r_s_ | r_0_ |
| --- | --- | --- | --- |
|  | a/Å | \|a\|/Å | a/Å |
| C_1 | 2,26 | 2.2714 ± 0.0008 | 2.280 ± 0.002 |
| C_3 | 0,057 | 0 | 0.056 ± 0.007 |
| C_4 | -1,38 | 1.376 ± 0.001 | -1.383 ± 0.008 |
| N | 0,907 | 0.907 ± 0.002 | 0.914 ± 0.002 |
| O | -1,79 | / | -1.815 ± 0.009 |
|  | b/Å | \|b\|/Å | b/Å |
| C_1 | -0,25 | 0.276 ± 0.006 | -0.244 ± 0.002 |
| C_3 | 0,788 | 0.777 ± 0.002 | 0.783 ± 0.004 |
| C_4 | 0,548 | 0.543 ± 0.003 | 0.548 ± 0.001 |
| N | -0,22 | 0.239 ± 0.007 | -0.235 ± 0.007 |
| O | -0,76 | / | -0.752 ± 0.006 |
|  | c/Å | \|c\|/Å | c/Å |
| C_1 | -0,17 | 0.204 ± 0.008 | -0.156 ± 0.001 |
| C_3 | -0,25 | 0.241 ± 0.006 | -0.243 ± 0.002 |
| C_4 | 0,183 | 0.179 ± 0.009 | 0.191 ± 0.001 |
| N | 0,386 | 0.361 ± 0.005 | 0.364 ± 0.001 |
| O | -0,17 | / | -0.171 ± 0.002 |

Table S17. Partial r_0_ structure of MAE *gG’T*. Calculated geometry (MP2/6-311++G**) and fitted parameters (in bold)

| Atom | Connectivity | | | Distance (Å) | Angle | Dihedral |
| --- | --- | --- | --- | --- | --- | --- |
| C_1 |  |  |  |  |  |  |
| N_2 | 1 |  |  | 1.46186 |  |  |
| C_3 | 2 | 1 |  | 1.46318 | **113.9 ± 0.5** |  |
| C_4 | 3 | 2 | 1 | 1.52134 | **109.2 ± 0.5** | **170.4 ± 0.1** |
| O_5 | 4 | 3 | 2 | 1.41699 | **110.9 ± 0.8** | -57.74 |
| H_6 | 5 | 4 | 3 | 0.96689 | 104.01 | 41.46 |
| H_7 | 4 | 3 | 5 | 1.09291 | 110.54 | -118.82 |
| H_8 | 4 | 3 | 5 | 1.10131 | 109.42 | 122.26 |
| H_9 | 3 | 2 | 4 | 1.09539 | 107.45 | -117.48 |
| H_10 | 3 | 2 | 4 | 1.10318 | 113.27 | 123.22 |
| H_11 | 2 | 1 | 3 | 1.01726 | 109.09 | -120.85 |
| H_12 | 1 | 2 | 3 | 1.09264 | 109.49 | 176.24 |
| H_13 | 1 | 2 | 12 | 1.09339 | 108.9 | 118.02 |
| H_14 | 1 | 2 | 12 | 1.10141 | 113.33 | -121.51 |

Table S18. Principal axis coordinates of heavy atoms in MAE *gG’G*

|  |  | r_e_ MP2 | r_e_ B3LYP | r_s_ | r_0_ |
| --- | --- | --- | --- | --- | --- |
|  |  | a/Å | a/Å | \|a\|/Å | a/Å |
| C_1 |  | -1,904 | -1,95 | 1.901(1) | -1.901(2) |
| N_2 |  | -0,965 | -0,976 | 0.966(2) | -0.980(1) |
| C_3 |  | -0,022 | -0,02 | 0.12(1) | -0.021(4) |
| C_4 |  | 1,128 | 1,15 | 1.133(1) | 1.132(3) |
|  |  | b/Å | b/Å | \|b\|/Å | b/Å |
| C_1 |  | -0,458 | -0,439 | 0.472(4) | -0.486(2) |
| N_2 |  | -0,054 | -0,072 | 0.03(6) | -0.018(2) |
| C_3 |  | 0,98 | 0,968 | 0.977(2) | 0.987(1) |
| C_4 |  | 0,324 | 0,344 | 0.291(6) | 0.3070(2) |
|  |  | c/Å | c/Å | \|c\|/Å | c/Å |
| C_1 |  | 0,436 | 0,435 | 0.418(4) | 0.4252(1) |
| N_2 |  | -0,609 | -0,592 | 0.610(4) | -0.610(2) |
| C_3 |  | -0,175 | -0,196 | 0.07(2) | -0.146(1) |
| C_4 |  | 0,583 | 0,566 | 0.573(3) | 0.585(1) |

Table S19. Partial r_0_ structure of MAE *gG’G*. Calculated geometry (MP2/6-311++G**) and fitted parameters (in bold)

| Atom | Connectivity | |  | Distance | Angle | Dihedral |
| --- | --- | --- | --- | --- | --- | --- |
| C_1 |  |  |  |  |  |  |
| N_2 | 1 |  |  | 1.463 |  |  |
| C_3 | 2 | 1 |  | 1.465 | **114.1(2)°** |  |
| C_4 | 3 | 2 | 1 | 1.525 | **109.9(2)°** | **80.3(2)°** |
| O_5 | 4 | 3 | 2 | 1.416 | **110.7(3)°** | 54.56° |
| H_6 | 5 | 4 | 3 | 0.968 | 103.79° | -39.30° |
| H_7 | 4 | 3 | 5 | 1.099 | 110.22° | -122.52° |
| H_8 | 4 | 3 | 5 | 1.093 | 110.09° | 118.59° |
| H_9 | 3 | 2 | 4 | 1.103 | 112.58° | -122.57° |
| H_10 | 3 | 2 | 4 | 1.094 | 107.95° | 117.53° |
| H_11 | 2 | 1 | 3 | 1.014 | 109.46° | -122.63° |
| H_12 | 1 | 2 | 3 | 1.092 | 109.31° | 175.30° |
| H_13 | 1 | 2 | 12 | 1.093 | 109.80° | 117.91° |
| H_14 | 1 | 2 | 12 | 1.102 | 112.95° | -121.33° |

Table S20. MP2/6-311**G(p,d) structure of the g'GG MAE conformer.

| *g'GG*  *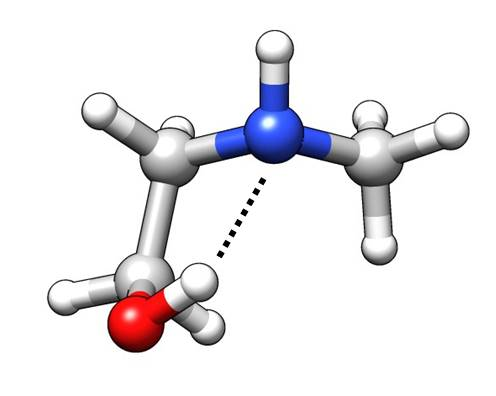* | Atom | a (Å) | b (Å) | c (Å) |
| --- | --- | --- | --- | --- |
|  | C**_1_** | -1.903 | -0.459 | 0.436 |
|  | N_2_ | -0.965 | -0.052 | -0.610 |
|  | C_3_ | -0.021 | 0.980 | -0.174 |
|  | C_4_ | 1.128 | 0.323 | 0.583 |
|  | O_5_ | 1.720 | -0.688 | -0.213 |
|  | H_6_ | 0.965 | -1.109 | -0.651 |
|  | H_7_ | 0.768 | -0.091 | 1.535 |
|  | H_8_ | 1.900 | 1.063 | 0.809 |
|  | H_9_ | -0.498 | 1.751 | 0.456 |
|  | H_10_ | 0.387 | 1.462 | -1.067 |
|  | H_11_ | -1.481 | 0.279 | -1.416 |
|  | H_12_ | -2.626 | -1.164 | 0.0179 |
|  | H_13_ | -1.365 | -0.972 | 1.237 |
|  | H_14_ | -2.449 | 0.389 | 0.878 |

Table S21. MP2/6-311**G(p,d) structure of the gG'G' MAE conformer.

| *gG'G*'  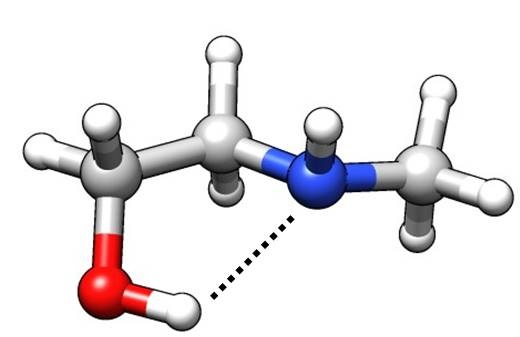 | Atom | a (Å) | b (Å) | c (Å) |
| --- | --- | --- | --- | --- |
|  | C**_1_** | 2.212 | -0.228 | -0.147 |
|  | N_2_ | 0.852 | -0.234 | 0.391 |
|  | C_3_ | -0.013 | 0.764 | -0.237 |
|  | C_4_ | -1.452 | 0.487 | 0.173 |
|  | O_5_ | -1.829 | -0.827 | -0.202 |
|  | H_6_ | -1.055 | -1.363 | 0.014 |
|  | H_7_ | -2.133 | 1.180 | -0.328 |
|  | H_8_ | -1.557 | 0.629 | 1.260 |
|  | H_9_ | 0.074 | 0.639 | -1.321 |
|  | H_10_ | 0.267 | 1.802 | 0.013 |
|  | H_11_ | 0.889 | -0.049 | 1.391 |
|  | H_12_ | 2.826 | -0.944 | 0.405 |
|  | H_13_ | 2.180 | -0.543 | -1.193 |
|  | H_14_ | 2.695 | 0.761 | -0.099 |

Table S22. MP2/6-311**G(p,d) structure of the g'GT MAE conformer.

| *g'GT*  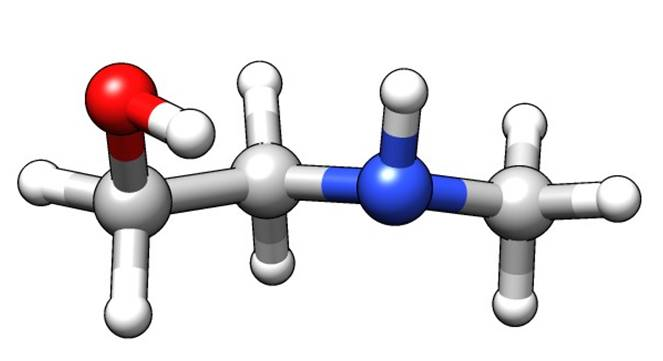 | Atom | a (Å) | b (Å) | c (Å) |
| --- | --- | --- | --- | --- |
|  | C**_1_** | 2.335 | -0.147 | -0.006 |
|  | N_2_ | 0.902 | -0.394 | -0.165 |
|  | C_3_ | 0.091 | 0.749 | 0.251 |
|  | C_4_ | -1.334 | 0.585 | -0.257 |
|  | O_5_ | -1.898 | -0.659 | 0.152 |
|  | H_6_ | -1.501 | -1.329 | -0.412 |
|  | H_7_ | -1.340 | 0.668 | -1.351 |
|  | H_8_ | -1.975 | 1.367 | 0.158 |
|  | H_9_ | 0.520 | 1.654 | -0.194 |
|  | H_10_ | 0.086 | 0.884 | 1.346 |
|  | H_11_ | 0.650 | -1.184 | 0.423 |
|  | H_12_ | 2.889 | -1.059 | -0.242 |
|  | H_13_ | 2.644 | 0.628 | -0.713 |
|  | H_14_ | 2.613 | 0.181 | 1.008 |

Table S23. MP2 6-311**G(p,d) structure of the tGT MAE conformer.

| *tGT*  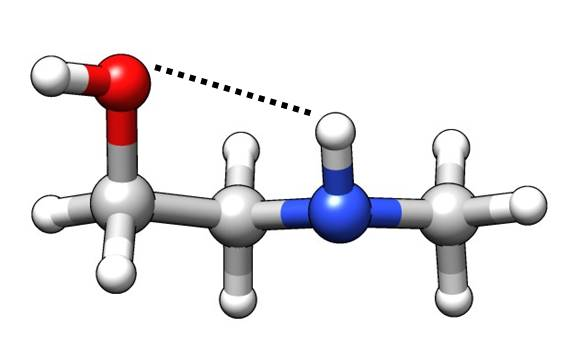 | Atom | a (Å) | b (Å) | c (Å) |
| --- | --- | --- | --- | --- |
|  | C**_1_** | 2.303 | -0.212 | 0.122 |
|  | N_2_ | 0.937 | -0.253 | -0.389 |
|  | C_3_ | 0.080 | 0.732 | 0.253 |
|  | C_4_ | -1.346 | 0.576 | -0.231 |
|  | O_5_ | -1.796 | -0.711 | 0.202 |
|  | H_6_ | -2.625 | -0.898 | -0.246 |
|  | H_7_ | -1.360 | 0.644 | -1.325 |
|  | H_8_ | -1.974 | 1.369 | 0.194 |
|  | H_9_ | 0.438 | 1.735 | -0.012 |
|  | H_10_ | 0.090 | 0.654 | 1.356 |
|  | H_11_ | 0.535 | -1.170 | -0.213 |
|  | H_12_ | 2.893 | -1.005 | -0.345 |
|  | H_13_ | 2.757 | 0.748 | -0.147 |
|  | H_14_ | 2.367 | -0.324 | 1.217 |

Table S24. MP2 6-311**G(p,d) structure of the gGT MAE conformer.

| *tG'G*'  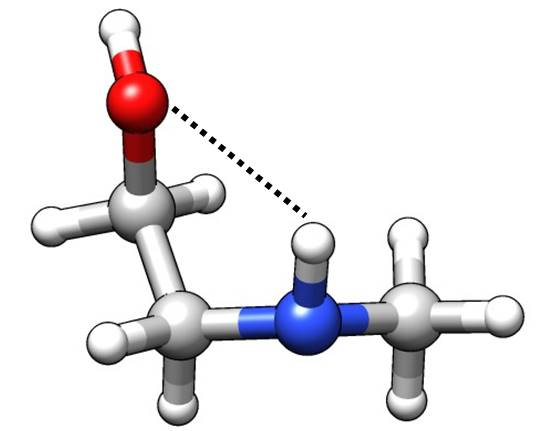 | Atom | a (Å) | b (Å) | c (Å) |
| --- | --- | --- | --- | --- |
|  | C_1_ | 0.036 | 1.019 | -0.054 |
|  | N_2_ | 1.108 | 0.201 | -0.605 |
|  | C_3_ | -1.105 | 0.256 | 0.612 |
|  | C_4_ | 1.783 | -0.632 | 0.384 |
|  | O_5_ | -1.619 | -0.655 | -0.359 |
|  | H_6_ | -2.332 | -1.152 | 0.051 |
|  | H_7_ | -1.875 | 0.971 | 0.932 |
|  | H_8_ | -0.748 | -0.285 | 1.498 |
|  | H_9_ | -0.381 | 1.620 | -0.868 |
|  | H_10_ | 0.469 | 1.709 | 0.681 |
|  | H_11_ | 0.719 | -0.390 | -1.334 |
|  | H_12_ | 2.614 | -1.154 | -0.096 |
|  | H_13_ | 2.199 | 0.014 | 1.164 |
|  | H_14_ | 1.142 | -1.384 | 0.867 |

Table S25. MP2 6-311**G(p,d) structure of the gG'G' MAE conformer.

| *gG'G*  '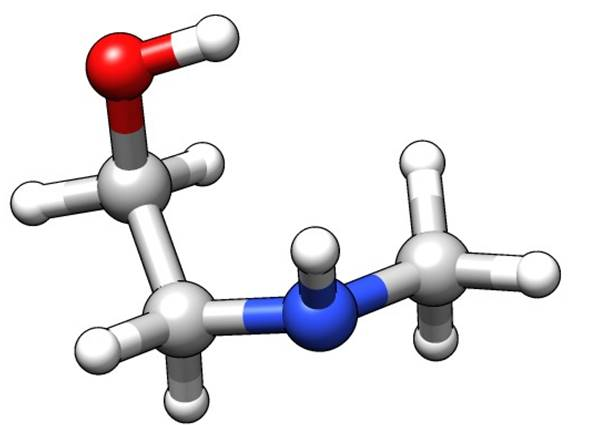 | Atom | a (Å) | b (Å) | c (Å) |
| --- | --- | --- | --- | --- |
|  | C_1_ | 0.041 | 1.035 | -0.032 |
|  | N_2_ | 1.144 | 0.251 | -0.575 |
|  | C_3_ | -1.111 | 0.246 | 0.592 |
|  | C_4_ | 1.762 | -0.673 | 0.367 |
|  | O_5_ | -1.711 | -0.657 | -0.333 |
|  | H_6_ | -1.081 | -1.366 | -0.485 |
|  | H_7_ | -1.900 | 0.940 | 0.892 |
|  | H_8_ | -0.777 | -0.286 | 1.492 |
|  | H_9_ | -0.358 | 1.648 | -0.847 |
|  | H_10_ | 0.446 | 1.715 | 0.730 |
|  | H_11_ | 0.856 | -0.226 | -1.422 |
|  | H_12_ | 2.621 | -1.151 | -0.109 |
|  | H_13_ | 2.128 | -0.104 | 1.228 |
|  | H_14_ | 1.102 | -1.468 | 0.752 |

Table S26. MP2 6-311**G(p,d) structure of the *tG'G'* MAE conformer.

| tG'G'  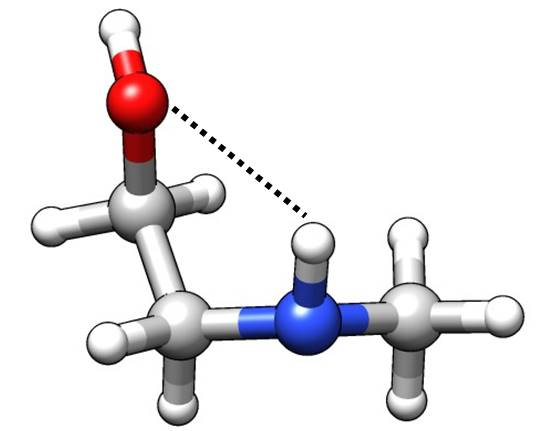 | Atom | a (Å) | b (Å) | c (Å) |
| --- | --- | --- | --- | --- |
|  | C_1_ | 0.036 | 1.019 | -0.054 |
|  | N_2_ | 1.108 | 0.201 | -0.605 |
|  | C_3_ | -1.105 | 0.256 | 0.612 |
|  | C_4_ | 1.783 | -0.632 | 0.384 |
|  | O_5_ | -1.619 | -0.655 | -0.359 |
|  | H_6_ | -2.332 | -1.152 | 0.051 |
|  | H_7_ | -1.875 | 0.971 | 0.932 |
|  | H_8_ | -0.748 | -0.285 | 1.498 |
|  | H_9_ | -0.381 | 1.620 | -0.868 |
|  | H_10_ | 0.469 | 1.709 | 0.681 |
|  | H_11_ | 0.719 | -0.390 | -1.334 |
|  | H_12_ | 2.614 | -1.154 | -0.096 |
|  | H_13_ | 2.199 | 0.014 | 1.164 |
|  | H_14_ | 1.142 | -1.384 | 0.867 |

Table27. MP2 6-311**G(p,d) structure of the *g'G'G*' MAE conformer.

| g'G'G'  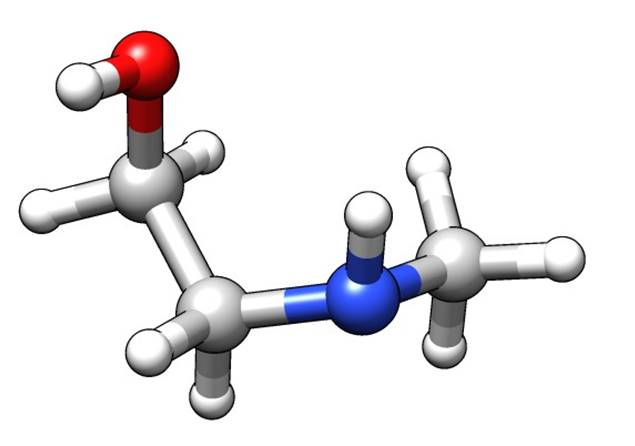 | Atom | a (Å) | b (Å) | c (Å) |
| --- | --- | --- | --- | --- |
|  | C_1_ | 0.047 | 0.996 | -0.048 |
|  | N_2_ | 1.105 | 0.177 | -0.625 |
|  | C_3_ | -1.095 | 0.232 | 0.624 |
|  | C_4_ | 1.850 | -0.598 | 0.364 |
|  | O_5_ | -1.716 | -0.682 | -0.278 |
|  | H_6_ | -2.149 | **-**0.157 | -0.958 |
|  | H_7_ | -1.824 | 0.944 | 1.033 |
|  | H_8_ | -0.725 | -0.380 | 1.449 |
|  | H_9_ | -0.372 | 1.618 | -0.850 |
|  | H_10_ | 0.498 | 1.679 | 0.683 |
|  | H_11_ | 0.689 | -0.458 | -1.300 |
|  | H_12_ | 2.660 | -1.132 | -0.139 |
|  | H_13_ | 2.300 | 0.091 | 1.085 |
|  | H_14_ | 1.250 | -1.334 | 0.919 |
